# Statistical framework to determine indel-length distribution

**DOI:** 10.1093/bioinformatics/btae043

**Published:** 2024-01-25

**Authors:** Elya Wygoda, Gil Loewenthal, Asher Moshe, Michael Alburquerque, Itay Mayrose, Tal Pupko

**Affiliations:** The Shmunis School of Biomedicine and Cancer Research, George S. Wise Faculty of Life Sciences, Tel Aviv University, Tel Aviv 69978, Israel; The Shmunis School of Biomedicine and Cancer Research, George S. Wise Faculty of Life Sciences, Tel Aviv University, Tel Aviv 69978, Israel; The Shmunis School of Biomedicine and Cancer Research, George S. Wise Faculty of Life Sciences, Tel Aviv University, Tel Aviv 69978, Israel; The Shmunis School of Biomedicine and Cancer Research, George S. Wise Faculty of Life Sciences, Tel Aviv University, Tel Aviv 69978, Israel; School of Plant Sciences and Food Security, George S. Wise Faculty of Life Sciences, Tel Aviv University, Tel Aviv 69978, Israel; The Shmunis School of Biomedicine and Cancer Research, George S. Wise Faculty of Life Sciences, Tel Aviv University, Tel Aviv 69978, Israel

## Abstract

**Motivation:**

Insertions and deletions (indels) of short DNA segments, along with substitutions, are the most frequent molecular evolutionary events. Indels were shown to affect numerous macro-evolutionary processes. Because indels may span multiple positions, their impact is a product of both their rate and their length distribution. An accurate inference of indel-length distribution is important for multiple evolutionary and bioinformatics applications, most notably for alignment software. Previous studies counted the number of continuous gap characters in alignments to determine the best-fitting length distribution. However, gap-counting methods are not statistically rigorous, as gap blocks are not synonymous with indels. Furthermore, such methods rely on alignments that regularly contain errors and are biased due to the assumption of alignment methods that indels lengths follow a geometric distribution.

**Results:**

We aimed to determine which indel-length distribution best characterizes alignments using statistical rigorous methodologies. To this end, we reduced the alignment bias using a machine-learning algorithm and applied an Approximate Bayesian Computation methodology for model selection. Moreover, we developed a novel method to test if current indel models provide an adequate representation of the evolutionary process. We found that the best-fitting model varies among alignments, with a Zipf length distribution fitting the vast majority of them.

**Availability and implementation:**

The data underlying this article are available in Github, at https://github.com/elyawy/SpartaSim and https://github.com/elyawy/SpartaPipeline.

## 1 Introduction

Insertions and deletions (indels) of short DNA segments are frequent mutational events, which were shown to constitute a large part of the genetic differences between closely related species (Britten 2002, Anzai *et al.* 2003, Wetterbom *et al.* 2006). Assumptions regarding the length distribution of indels are embedded either explicitly or implicitly in various bioinformatics applications, most notably in alignment methods. It was previously shown that the assumed indel distribution can substantially alter the resulting alignments (Lunter *et al.* 2008). The correct placement of indels was previously shown to affect the accuracy of additional computational tasks, such as ancestral sequence reconstruction and the inference of selective patterns (Ashkenazy *et al.* 2012, Vialle *et al.* 2018), which rely on the alignment as input.

In both protein and DNA alignments, the most frequent indel event is a deletion or an insertion of a single amino acid or a single base-pair, respectively. The frequency of events monotonically declines with the size of the indel (Pascarella and Argos 1992, Benner *et al.* 1993, Golenberg *et al.* 1993, Gu and Li 1995, Qian and Goldstein 2001). Two main distributions were proposed to characterize indel lengths, geometric and Zipf. Due to its computational simplicity, the geometric distribution is assumed in the most popular alignment algorithms. For example, the classic algorithms of Needleman and Wunsch (1970) and Smith and Waterman (1981) utilize a linear-gap penalty, which implicitly assumes that indel lengths are distributed geometrically. However, it was previously claimed that the Zipf distribution better fits empirical datasets, both for proteins (Benner *et al.* 1993, Fan *et al.* 2007) and non-coding segments (Saitou and Ueda 1994, Gu and Li 1995, Fan *et al.* 2007). Gu and Li (1995) studied the length distribution of deletions and insertions within human and rodent pseudogenes and found only small differences between indels. Fan *et al.* (2007) analyzed coding and non-coding indels in 18 mammalian genomes and found differences in length distributions both among species and between indels. Of note, the inferred Zipf parameter was smaller than two in all these studies, which means that no moments of the distribution are defined, including its mean and standard deviation. Moreover, Qian and Goldstein (2001) showed that the frequency of longer indels declines rapidly. Together, these results suggest that the indel-length distribution is not a pure Zipf. Hence, a truncated Zipf distribution, in which the maximum size of indels is constrained, was used in some studies (Cartwright 2006, Loewenthal *et al.* 2021).

Notably, the above-mentioned studies used gap-counting heuristics for inferring indel events. These heuristics suffer from several limitations. First, it is assumed that a continuous stretch of gap characters (termed hereby “gap block”) in the alignment corresponds to a single indel event. This is not always the case, as multiple indel events can contribute to a single gap block. Second, in previous approaches, overlapping gaps were usually not used for inference as they are hard to interpret. Third, gaps are derived from an alignment, and alignments are seldom free of errors. As discussed above, alignment algorithms implicitly assume an indel-length distribution. This assumption may introduce biases to the inferred alignment, and more specifically, it may affect the distribution of gap lengths. Further, even in simulations, where the indel-length distribution is known and matches the alignment assumptions, many of the alignment errors are related to the placement of gaps (Lunter *et al.* 2008).

One way to minimize the effects of overlapping indels and alignment uncertainty on the inference of indel dynamics is to analyze closely related sequences. The benefit of such an approach is that the alignment among these sequences is often highly reliable due to the low number of substitutions and indel events. In these cases, the frequency of overlapping indels is small, and thus each gap block typically corresponds to a single indel event. This approach is inapplicable when the goal is to characterize indel event dynamics among highly diverged genomes. Additionally, because the total number of indel events is relatively small when analyzing closely related sequences, such efforts must base their inference on large segments of chromosomal DNA, e.g. within human populations or between closely related primates (Tanay and Siggia 2008, The 1000 Genomes Project Consortium *et al.* 2015).

While it is possible to infer indel dynamics by analyzing large genomic regions in closely related genomes, this kind of analysis will fail to capture the spatial variation in indel patterns among genomic regions. For example, indels are more common in introns and other non-coding regions than in protein-coding genes. Variation in the number of indel events among proteins is also common, because similar to substitution events, the evolutionary selective forces, which dictate indel dynamics depend on the nature of the analyzed protein, e.g. indels may destabilize the protein’s 3D structure in one protein but have negligible effect on the structure in another. It was previously shown that even within introns, deletions are more common in the middle of the intron compared to its borders with exons due to a phenomenon we have previously termed border-induced selection (Loewenthal *et al.* 2022). Thus, each of the two previous approaches to infer indel dynamics suffers from some limitations: gap-counting methods lack statistical robustness and are sensitive to alignment uncertainty when diverged sequences are analyzed and methodologies that base their inference on large segments of closely related chromosomal DNA segments are unable to infer indel dynamics across diverged sequences and cannot provide an adequate description of locus-specific indel dynamics. Probabilistic-based methods offer the possibility to analyze indel dynamics among specific proteins or genes across a set of diverged species, within the framework of a reliable statistical framework.

Despite the evolutionary significance of indels, and in stark contrast to substitutions, there are only few probabilistic models of indels. A reason for this scarcity is that indels often span over multiple sites, which hamper the usage of likelihood-based methods that, for computational tractability, usually assume independence among sites (Cartwright 2005, Fletcher and Yang 2009). To determine which length distribution better fits an empirical dataset, Cartwright (2009) introduced two hidden Markov models (HMMs) between pairs of sequences. The first HMM assumes that indel length follows a geometric distribution, while the second HMM assumes a Zipf distribution. This HMM-based methodology showed that Zipf provided a better fit to orthologous introns of primates and rodents. This pioneering work alleviated many of the problems in gap-counting methods, yet it has some limitations. First, this method is applicable only for two sequences and does not consider the evolutionary distance between the sequences. Second, this method is computationally demanding, and thus it cannot be easily extended to more complex evolutionary models. For example, the method utilized the simple K2P substitution model (Kimura 1980), and more complex substitution models were not considered due to memory issues. Third, it does not allow a rigorous model selection scheme on a wide range of model parameters. Finally, the HMMs ignored overlapping indels and did not distinguish between indels.

Approximate Bayesian computation (ABC) is a statistical procedure for inferring model parameters. ABC is commonly applied when it is challenging to compute the likelihood, as this procedure bypasses the need to explicitly calculate likelihood. The ABC procedure, which was first developed in the field of population genetics (Beaumont *et al.* 2002), has been since utilized successfully in many other studies (Przeworski 2003, Tallmon *et al.* 2008, Kuhlwilm *et al.* 2019, Moshe *et al.* 2022). Our group had previously utilized ABC for inferring the rates and length parameters of indels (Karin *et al.* 2017, Loewenthal *et al.* 2021). In these studies, a single length distribution was assumed (truncated Zipf).

In this study, we develop evolutionary models that allow for various indel-length distributions. We develop rigorous model selection schemes to determine which length distribution best fits a given empirical dataset and applied posterior predictive *p*-value tests to study how well the various models fit empirical datasets. Using simulations, we demonstrate that our model selection scheme is highly accurate. We apply our developed methodology to 67 intron datasets from YIDB (Lopez and Séraphin 2000) and 416 protein datasets from EggNOG (Huerta-Cepas *et al.* 2019). We show that while different datasets are characterized by different length distributions, the Zipf distribution fits most datasets. Using the predictive *p*-value test we also show that some empirical datasets do not fit to any of the suggested models and discuss reasons for such discrepancy. Furthermore, we show that even after removing datasets that exhibit poor fit to all models considered, the percentage of datasets assigned to each length distribution remains roughly the same with 74% of the datasets assigned to the Zipf distribution.

## 2 New approaches

### 2.1 Indel-length distribution classification outline

Our model selection scheme determines for a given empirical dataset the best-fitting indel model among a set of competing models, which differ in their assumptions regarding the shape of the indel-length distribution. In the following, we use the terms model selection and classification interchangeably, as we match (classify) each empirical dataset to one of the indel models, i.e. the best-fitting model for this dataset. The input for this methodology is the set of aligned sequences and their associated rooted phylogenetic tree with branch lengths measured in number of substitutions per site.

The indel-length distribution classification scheme is composed of the following parts: (i) specifying a set of hypotheses regarding the different indel-length distributions. In this work, we focus on selecting from three distributions: Zipf, geometric, and Poisson; (ii) ABC is a Bayesian inference scheme, and as such specifying the priors of all parameters are required. We assume equal prior probabilities for all three alternative length distributions. We also set priors for the parameters of each length distribution. Likewise, we also set priors for the other indel parameters that are shared among all models; (iii) simulating numerous multiple sequence alignments (MSAs) by repeatedly sampling an indel model and its associated parameters from the prior, and using this model to simulate an MSA along the input tree; (iv) extracting summary statistics from the resulting simulated MSAs; (v) accounting for the alignment errors by correcting the computed summary statistics using a machine-learning algorithm; (vi) extracting summary statistics from the empirical MSA and calculating the distances between the corrected simulated MSAs and empirical MSA summary statistics; and (vii) classifying the indel-length distribution of the empirical MSA according to the distribution obtained by the simulated MSAs. Below, we elaborate on each of these parts.

### 2.2 The indel-length distributions

We selected three candidate length distributions: Zipf, geometric, and Poisson. Zipf was reported in previous studies, which applied gap-counting techniques, as the most fitting distribution (Benner *et al.* 1993, Saitou and Ueda 1994, Gu and Li 1995, Fan *et al.* 2007); The geometric distribution is assumed in prevailing alignment software (Edgar 2004, Katoh and Standley 2013). In addition, we chose the Poisson distribution as a negative control. Of note, we truncated each of the distributions at a length of 150, as we study micro indels. Each of these distributions has different parameters, thus, we adjust their prior distributions to make sure that we sample from the same range of mean lengths. We set the parameters that control the prior range so that the mean indel length is between 1.5 and 25, which allows for a realistic range of indel lengths in our simulated MSAs (Jiang *et al.* 2015, Loewenthal *et al.* 2021). We first draw the log base 10 of the total rate, which is the sum of both the insertion and deletion parameters, then, we draw the ratio of the two rates. The priors for both indels are the same regardless of the assumed length distribution. In our experience, these priors provide alignments that are not too long (excessive insertion rate), nor too short (excessive deletion rate). The priors are specified in Table 1.

**Table 1.** Model priors: (a) Priors that are shared among all models; (b) priors that are specific to each distribution type.^a^

(a)		
Parameters	Priors	
	Lower limit	Higher limit
Root length	0.8 × SSA	1.1 × LSA
log_10_(Total rate)	−4	−1
log_10_(Rate ratio)	−1	1
Truncation	150	150

(b)						
Length distribution	Zipf		Geometric		Poisson	
Prior limits	Lower	Higher	Lower	Higher	Lower	Higher
Length parameter	1.03	2.78	0.04	0.66	1.5	25
Mean length	25	1.5	25	1.5	1.5	25

a SSA and LSA denote the shortest and longest sequence lengths in the alignment, respectively. Total rate and rate ratio are the sum and ratio of the insertion and deletion rates, respectively. All parameters are uniformly distributed between the lower and upper limits.

Additionally, we ran all models under two alternatives. In the first variant, we assumed that indels follow the exact same distribution, i.e. both their shape and their rate parameters are identical. We have previously termed this model SIM, for simple indel model. In the second variant, previously termed RIM (for rich indel model), the indel distribution family is assumed to be the same for indels (e.g. both indels are geometrically distributed), but parameters are sampled from the prior independently for indels, such that the rate and length distribution parameters of indels are different. We have previously shown that some datasets are better described by SIM, while others by RIM (Loewenthal *et al.* 2021). Thus, given an empirical dataset, we compared the fit of six alternative indel models (a SIM and a RIM version for each of the three length distributions).

### 2.3 Simulator

To simulate MSAs, we sample the parameters for insertion and deletion rates, the parameters governing the length distribution of indels, and the root sequence length from the specified priors. We proceed by traversing along each of the branches of the provided phylogenetic tree in preorder, using the rates of indels to determine the waiting time until an event occurs (Gillespie 1977). Assuming an event had occurred, its length is determined by sampling from the appropriate length distribution. Next, we draw uniformly the starting position of the event, correcting for possible biases in the edges of the sequences (Karin *et al.* 2017, Loewenthal *et al.* 2021). We proceed until all the sequences at the leaves of the tree have been generated. The output of the simulation is termed the “true” MSA.

### 2.4 Summary statistics

We define 27 summary statistics extracted from the true MSA. These summary statistics include general MSA characteristics, such as the longest and shortest sequence lengths (excluding gaps), and more gap specific features, e.g. the number of sites, which contain a single gap across all sequences. All 27 summary statistics are detailed in Supplementary Table S1. Summary statistics 13–27 are devoted to counting gap blocks of length one, two, three, four, or more. These summary statistics should help differentiate between various length distributions. For example, consider a dataset that evolved under a Poisson length distribution with a mean length of three for both indels. It is likely that gaps of length three would be more frequent than gap of length one. This is not true for both Zipf and geometric distributions, as they are monotonically decreasing.

### 2.5 Alignment correction

The MSAs generated by our simulator are accurate, since the simulator keeps track of all insertion and deletion events along the phylogenetic tree. In contrast, the empirical MSAs that are given as input are inferred by an alignment program and may contain errors, which can bias our inference and can lead to erroneous classifications. One way to correct this bias would be to realign the simulated sequences using the same alignment program that was used to align the empirical datasets, e.g. MAFFT (Katoh and Standley 2013). However, running an alignment program on each of the ABC simulations is computationally infeasible. Instead, we developed the following approach to transform the computed summary statistics by predicting the bias of a given dataset. First, we generate and align 500 simulations for each dataset. We then use a machine-learning algorithm (see Supplementary Methods) to learn how the summary statistics of the true alignment were distorted following the inferred alignment. The summary statistics of the remaining simulations are transformed accordingly.

### 2.6 Classification

To classify a given dataset, we simulate 500 500 MSAs for each of the six models (a total of 3 003 000 simulations for each empirical dataset) and compute the summary statistics for each simulated alignment. We use the machine-learning algorithm on the first 500 simulated MSAs, per model, to learn the bias transformation of the summary statistics and apply it on the entire set of simulated MSAs. We then measure the distance between each of the corrected summary statistics vectors of the simulated MSAs to the summary statistics vector of the empirical MSA. The distance between the summary statistics vectors is calculated using the Mahalanobis distance (Mahalanobis 1936), as some of the summary statistics are correlated. Then, we select the 100 simulations closest to the empirical dataset and classify the empirical indel-length distribution according to the distribution that is most frequently selected. For example, if among the chosen simulations, 40, 30, 20, 8, 2, and 0 are derived from Zipf-RIM, Zipf-SIM, geometric-RIM, geometric-SIM, Poisson-RIM, and Poisson-SIM distributions, respectively, we classify this dataset as Zipf-RIM. Because in this work, we are mainly interested in the shape of the distribution, we group the SIM and RIM models, and thus, 70 of the 100 simulations support the Zipf distribution. According to ABC theory, in this example, 70% is also an estimate of the posterior support for the Zipf distribution (Loewenthal *et al.* 2021).

### 2.7 Posterior predictive *p*-values

The above model selection procedure infers the relative fit of the six examined models to the data. Thus, it can only infer which model best fits those data, but it cannot infer whether the best-fitting model provides an adequate representation of the evolutionary dynamics (Ingvarsson 2008, Francois and Laval 2011). In the case of indel models, model inadequacy can point to cases in which all the considered indel models fail to capture the observed gap patterns in the empirical dataset analyzed. This can stem from oversimplified underlying assumptions, such as the uniformity of the indel parameters along the sequence (lack of spatial variation), and along the phylogeny (lack of heterotachy). In some datasets, these assumptions might not be reasonable. For instance, if highly diverged sequences are analyzed and the selective forces dictating indel dynamics have changed during the course of evolution.

We developed model adequacy tests, based on posterior predictive *p*-value simulations, to assess the absolute fit of the selected model to empirical data. Following Ingvarsson (2008) and Francois and Laval (2011), we assume that the adequacy of the model is related to how the empirical summary statistics resemble the simulated summary statistics. To this end, we choose the 50 simulations of the selected model that were closest to the empirical MSA in terms of their summary statistics. We then resample uniformly the indel parameters of these 50 simulations and generate 10 000 simulations, from which the summary statistics distributions are generated. Next, we determine the percentile rank of each summary statistic of the empirical MSA in the corresponding simulated distribution. Empirical summary statistic with a posterior predictive *p*-value outside the [0.025, 0.975] interval may point to model inadequacy. Since there are 27 summary statistics, we also introduce a single metric that summarizes the 27-posterior *p*-values. We call this metric adequacy match (AM), and it is defined as the percentage of summary statistics that have their posterior predictive *p*-value inside the [0.025, 0.975] interval. Getting an AM score of 100% would mean that the model generated MSAs that perfectly captured all summary statistics found within the empirical dataset.

## 3 Results

### 3.1 Performance on simulated data

We first quantified the performance of our ABC-based classification scheme on simulated data whose parameters were based on 483 empirical MSAs. For example, we used a phylogenetic tree inferred from 31 sequences taken from the EggNOG database (ENOG503HQ7D). We simulated using each of the three indel-length distributions 100 MSAs (50 for each model variant, i.e. 50 SIM models in which the parameters are indels are the same, and 50 RIM models in which separate sets of parameters are used for indels). We ran our classification scheme and measured its accuracy. For this dataset the classification accuracy was 94%, 99%, and 93% for the Zipf, Poisson, and geometric models, respectively (Table 2).

**Table 2.** Confusion matrix of the simulations derived using the phylogeny of the EggNOG dataset ENOG503HQ7D (Supplementary Fig. S4).

		Classification		
		Zipf	Geometric	Poisson
True distribution	Zipf	94	4	2
	Geometric	3	93	4
	Poisson	0	1	99

To detect which features contribute most to the classification accuracy, we repeated this analysis, each time excluding one of the features. As expected, the features whose exclusion reduced the accuracy the most were those related to the frequency of indels of certain lengths. Specifically, the feature specifying the number of gaps in the alignment of length higher than three was the most important for classification (given all other features), followed by the total number of gaps, and the number of gaps of length three (see Supplementary Table S2).

We also evaluated the impact of model parameters on classification accuracy. Specifically, we repeated the above example, however, this time using simulations with extreme indel rate parameters. Extremely high (low) indel rates were defined as the upper (lower) 0.01% of the prior distribution of the sum of the insertion and deletion rates. The classification accuracy was robust to these extreme rates (Supplementary Fig. S1). However, when sampling extreme length parameters, many Poisson simulations were classified as geometric and *vice versa*. Yet, the classifier could correctly distinguish between Zipf and the other distributions (Supplementary Fig. S1).

We repeated this analysis for all 416 EggNOG and 67 YIDB datasets used in this study. The average accuracy across all datasets and three models was 98.3%, the minimum accuracy for a single dataset was 91.6%, and for 57.7% of the datasets, our model selection scheme had an accuracy of 99% or higher (see Supplementary Fig. S2).

### 3.2 Analysis of empirical data

**Figure 1.**
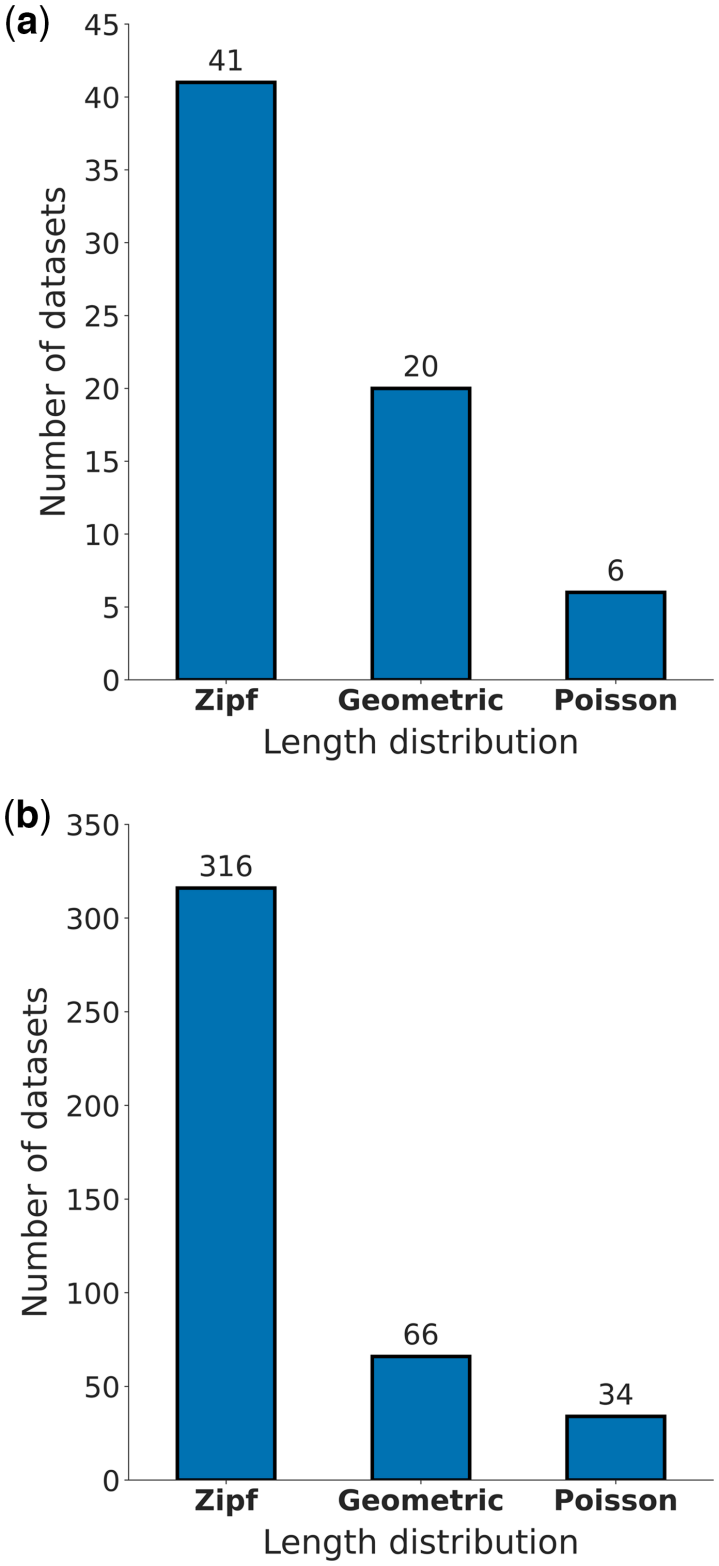
Histogram of classified length distributions: (a) YIDB datasets; (b) EggNOG datasets.

To learn which indel distribution characterizes empirical genomic data, we ran the classification scheme on a curated sample of 67 nucleotide datasets of yeast introns taken from YIDB (Lopez and Séraphin 2000) and 416 protein datasets from the EggNOG database (Huerta-Cepas *et al.* 2019). Our results indicated that among yeast introns most datasets follow the Zipf distribution, with 61.19%, 29.85%, and 8.96% of the datasets classified to the Zipf, geometric, and Poisson distributions, respectively. This trend was even more pronounced in the analyzed protein EggNOG alignments, with 75.96% of the datasets classified to the Zipf distribution and only 15.87% and 8.17% to the geometric and Poisson, respectively (Fig. 1). The posterior distributions of all model parameters span a wide range of values, indicating that these empirical data represent a spectrum of alignment characteristics (Supplementary Fig. S3).

**Figure 2.**
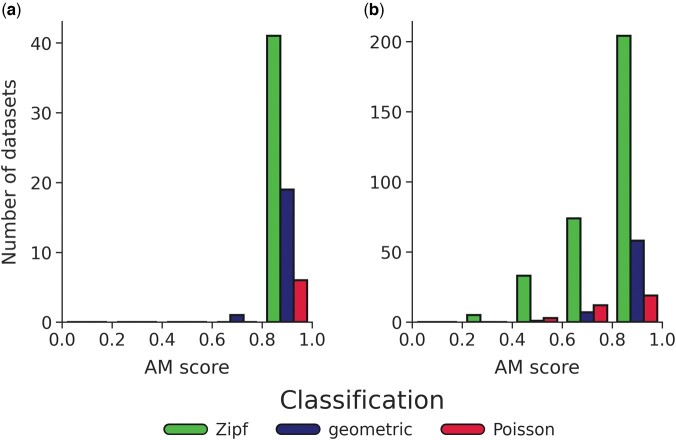
AM score histogram grouped by classified indel-length distribution, within five different AM score intervals (each of width 0.2). (a) YIDB datasets. (b) EggNOG datasets. In each bin, the left-most, middle, and right distributions are Zipf, geometric, and Poisson, respectively.

We next applied the model adequacy test on the selected model and the empirical data. To this end, we computed the AM score for each of the empirical datasets. This score quantifies the fraction of summary statistics, derived from the empirical data, which are within the range of values of the summary statistics generated by repeated parametric simulations. The histograms of the AM scores for YIDB and EggNOG are shown in Fig. 2a and b, respectively. For the YIDB dataset, the mean AM scores are 0.960, 0.975, and 0.932 for datasets classified as Zipf, geometric, and Poisson, respectively, while for EggNOG the corresponding means are 0.810, 0.921, and 0.820. The differences in the mean AM scores between the datasets that were classified as Zipf and those classified as geometric were statistically significant in the EggNOG dataset (*p *<* *0.01, independent samples *t*-test). Thus, even though Zipf was selected for more datasets, those datasets exhibited a lower absolute fit than those selected as geometric.

**Figure 3.**
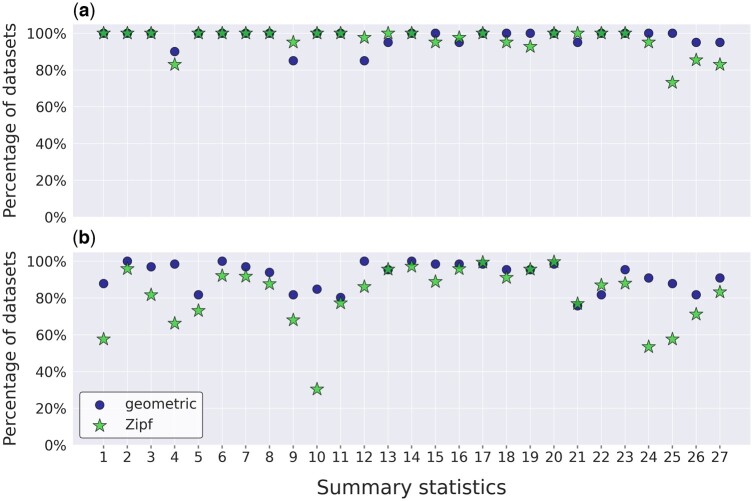
Summary of the posterior predictive analysis for each of the summary statistics obtained for: (a) the YIDB datasets; and (b) the EggNOG datasets. Dots and stars represent datasets that were classified as geometric and as Zipf, respectively. The *Y* axis represents the percentage of dataset for which the posterior predictive *p*-value was between 0.025 and 0.0975. The list of summary statistics is available in Supplementary Table S1.

Next, we searched for summary statistics that consistently exhibit low posterior *p*-values, i.e. their distribution in simulated datasets substantially differs from their distribution in empirical datasets. Finding such summary statistics may point to attributes of the empirical data that the model fails to capture. To this end, for each dataset and its classified distribution type, we examined the posterior *p*-value of each summary statistic, and tested whether it is within the [0.025, 0.975] interval (Supplementary Fig. S5). Figure 3 shows, for each summary statistics, the percentage of datasets for which the *p*-value is within this interval. Of note, we limited this analysis to datasets which were classified as Zipf and geometric, as there were not enough datasets that were classified as Poisson to draw conclusions regarding the fit of this model. The overall performance of both length distributions in the YIDB datasets was similar. For all but one summary statistics, the percentage of datasets with a posterior predictive *p*-value within the range [0.025, 0.975] was above 80%. The percentages were substantially lower in the EggNOG dataset, with 10 summary statistics having <80% of datasets within that range, including four summary statistics lower than 60%. Surprisingly, for the EggNOG datasets, the adequacy of datasets classified to have a geometric distribution was far better than Zipf (Fig. 3). The summary statistics for which the percentage of datasets was lowest for the Zipf distribution were the “average unique gap length” (30%) and the “number of positions within the MSA that contain no gaps” (53%). We propose the following explanation for these results: the Zipf distribution has more chance to generate very long indels, and thus MSAs in which missing data were coded as gap characters were classified as Zipf. These datasets are characterized by low posterior predictive *p*-values. As a control, we performed this analysis on simulated datasets. In these datasets, the model used to generate the data was the same as that assumed in the inference, and thus we expected that most datasets would have non-extreme posterior *p*-values. This was indeed the pattern observed (Supplementary Fig. S6).

When comparing the absolute fit of the selected models that were classified as Zipf, the EggNOG protein datasets scored significantly lower than the YIDB intron datasets (*t*-test between AM scores; *p*-value <0.01). This was not the case when comparing datasets that were classified as geometric (*p*-value >0.01). Thus, some of the model assumptions might not be adequate for some of the EggNOG datasets. We searched for such assumptions by inspecting some of the worst performing datasets, and visualized their MSAs. The dataset with the lowest AM score (ENOG504MRKW) was composed of a set of orthologous primate proteins in which some of the sequences were included in the MSA despite annotation errors, leading to unusually large gaps spanning most of the MSA (Supplementary Fig. S7). These types of issues were prevalent in all low scoring datasets we inspected. We hypothesized that extreme gap block sizes are associated with low AM scores. A correlation test between the AM scores and the average sizes of unique gaps revealed a significant correlation of −0.66 (*p*-value <10^−52^), supporting this hypothesis.

It is possible that the results above regarding the percentage of datasets that are classified as Zipf versus geometric are affected by such problematic datasets similar to the one shown in Supplementary Fig. S7. To test this hypothesis, we report the classification results for only those datasets with AM higher than 0.8. The results remained similar, i.e. the vast majority of the datasets were still classified as Zipf, but with a slight increase in the proportion of datasets classified as geometric (Supplementary Fig. S8). This result corroborates our previous hypothesis that problematic datasets with very long indels were classified as Zipf.

## 4 Discussion

The aim of this work was to identify the distribution that best describes indel dynamics as seen in MSAs of empirical data. We employed ABC, a robust simulation-based statistical method, to select between alternative models. The absolute fit of the models to the data was also tested using posterior predictive *p*-values. The classification was performed on hundreds of protein datasets, sampled from EggNOG, and dozens of intron datasets, sampled from YIDB. In contrast to the notion that the length distribution of indels is best explained by a Zipf distribution, our results suggest that the best-fitting length distribution is dataset dependent, with some datasets best described by a Zipf distribution and others, by a geometric distribution. When missing data or other annotation errors are common, the dataset may be classified as Zipf to capture the existence of very long gap blocks. This is true for both coding and non-coding datasets. The parameters of the various distributions also varied across datasets. This variability among datasets may stem from various factors, including selective forces, mutation patterns, and differences between the groups from which the datasets were sampled. The low AM scores obtained for some cases suggest that our inference scheme can also identify MSAs whose gaps pattern reflects missing data and annotation errors rather than true indel events.

Understanding the underlying dynamics of indels, and devising better models, is important for many bioinformatics applications. Most notably, it is important for improving alignment accuracy (Lunter *et al.* 2008). The most popular alignment methods utilize a fast and simple scoring method called affine-gap penalty, which implies a geometric indel-length distribution (Altschul and Erickson 1986). Unfortunately, gap penalty scores corresponding to a Zipf distribution are more complex to derive. Cartwright (2006) approximated this penalty by ignoring overlapping events. The obtained gap penalty was a combination of logarithmic and affine terms. Cartwright showed on simulated sequences that this gap penalty results in better pair-alignments compared to both affine and log penalties. Our results here indicated that incorporating such methodologies should prove beneficial to most empirical sequence datasets. Promising future directions should focus on the application of similar techniques to alignments composed of more than two sequences.

Recently, a novel penalty-free alignment method was developed (Maiolo *et al.* 2018, 2020). This method reduced the computational time by relying on a Poisson indel process (PIP) (Bouchard-Côté and Jordan 2013) that is closely related to the Markov process model of TKF91 (Thorne *et al.* 1991). Of note, both PIP and TKF91 allow only indels of length one. Interestingly, this novel alignment approach showed comparable accuracy to Prank (Löytynoja 2014) and MAFFT (Katoh and Standley 2013), both of which allow indels of arbitrary length. Relaxing the indel size assumption in PIP and introducing Zipf into the model may further improve its accuracy. In addition, we recently developed a novel method for aligning sequences based on natural language processing deep-learning architectures (Dotan *et al.* 2023: https://openreview.net/forum?id=8efJYMBrNb). The strength of this approach is that it is often time easier to simulate complex evolution phenomena rather than model them or calculate their corresponding penalty. However, the accuracy of this method relies on simulated MSAs, thus, simulating under realistic indel models is crucial.

Many computational tools rely on MSAs as input; therefore, alignment inaccuracies may propagate and affect downstream results. For example, indel sizes and locations are critical to the inference scheme of ancestral sequence reconstruction (Liberles 2007). In FastML, a likelihood-based ancestral sequence reconstruction tool, indels are encoded using the simple and heuristic indel coding method (Simmons and Ochoterena 2000). It then computes the posterior probability of indels at each site in every node of the phylogenetic tree using a two-states Markov process (Cohen *et al.* 2008). Ideally, the indel models suggested in this work as well as the model selection scheme would be integrated in ancestral sequence reconstruction algorithms to improve the accuracy of inference.

Elucidating indel dynamics is also important for understanding the length distribution of neutral segments (Ogata *et al.* 1996, Moriyama *et al.* 1998). We have recently proposed that a phenomenon termed border-induced selection may partially explain the existence of very long introns within the human genome (Loewenthal *et al.* 2022). Accordingly, in a neutral sequence that is bordered by functional segments, deletions that encompass these functional segments are rejected. We showed that the magnitude of border-induced selection depends on the variation of the deletion length distributions. For a given mean, the truncated Zipf distribution has a higher variation than the geometric distribution, and thus longer neutral sequences are expected if the deletion lengths distribution follows the Zipf distribution.

In practice, our method, applying the proposed posterior predictive *p*-value test, can be directly utilized to determine whether standard indel models, as proposed in this study, adequately fit a given empirical dataset. In those cases, where the models are rejected, future data inspection is recommended. For example, such an approach can detect cases of extremely long indels, which correspond to annotation problems. In addition, our methodology should be used whenever simulated MSAs are used, as it allows the simulated MSAs to better reflect empirical datasets. As a case in point, simulated MSAs have recently been employed to train deep-learning algorithms for sequence alignment, as these simulated MSAs were generated following indel parameters that were inferred from empirical datasets (Dotan *et al.* 2023).

In theory, the inferred indel model parameters could have been used to determine gap penalty parameters. Specifically, the cost of indels of a specific length should vary depending on whether the dataset was classified as geometric or Zipf, and in each case, according to the parameter of the inferred distribution. Unfortunately, none of the currently popular alignment programs allow a Zipfian distribution. Thus, regardless of the gap distribution inferred to best fit an empirical dataset, researchers can only use programs that implicitly assume a geometric distribution. In addition, even if an empirical dataset is classified as geometric, the mapping between the parameters of the geometric model and the optimal gap penalty parameters is unknown. Future applications could apply machine-learning approaches to learn such a mapping.

## 5 Source and data availability

### 5.1 Empirical datasets

The datasets analyzed were taken from YIDB (Lopez and Séraphin 2000) and EggNOG (Huerta-Cepas *et al.* 2019). Only alignments with total branch lengths higher than one were retained, as it was previously shown in Loewenthal *et al.* (2021) that the accuracy of the ABC scheme is tightly correlated with branch lengths. Additionally, since our method uses the gaps in the alignments to generate summary statistics, we filtered all MSAs containing <20 unique gaps. All datasets that remained after filtering were unaligned and realigned using MAFFT. The 67 MAFFT-based YIDB MSAs that remained after filtering are available at https://github.com/elyawy/SpartaPipeline.

### 5.2 Source code

Our indel simulator was written in C++ and Python and can be found at https://github.com/elyawy/SpartaSim. The inference scheme and adequacy scripts written in Python are available at https://github.com/elyawy/SpartaPipeline.

## Supplementary Material

btae043_Supplementary_Data
