# Supplementary material for: Statistical framework to determine indel-length distribution
Source: Bioinformatics. 2024 Jan 25;40(2):btae043. doi: 10.1093/bioinformatics/btae043 (PMC10868340; doi:10.1093/bioinformatics/btae043)
Supplement: btae043_Supplementary_Data [file btae043_supplementary_data.docx]

**Statistical framework to determine indel length distribution**

**Supplementary materials**

Elya Wygoda^1*^, Gil Loewenthal^1*^, Asher Moshe^1^, Michael Alburquerque^1^, Itay Mayrose^2^, and Tal Pupko^1,^†

^1^ [The Shmunis School of Biomedicine and Cancer Research](https://en-lifesci.tau.ac.il/lp-en-mcbb), George S. Wise Faculty of Life Sciences, Tel Aviv University, Tel Aviv 69978, Israel.

^2^ School of Plant Sciences and Food Security, George S. Wise Faculty of Life Sciences, Tel Aviv University, Tel Aviv 69978, Israel.

* These authors contributed equally.

† To whom correspondence should be addressed:

Tal Pupko, Tel: +972 3 640 7693; Fax: +972 3 642 2046; E-mail: talp@tauex.tau.ac.il

Table S1 All 27 summary statistics used by the inference scheme to classify a given dataset.

| **#** | **Summary statistic description** | **Highlighted on example MSA** | **Value** |
| --- | --- | --- | --- |
|  | The average length of gaps in the MSA:  $\frac{\text{number of '-' characters}}{\text{total number of gaps}}$ | -AAAAAAAAAAAAAAAAA---AAAAAAAAAAA-----  A----AA-AA-AAAA--A---A---A-----AAAAAA  A---AAA-AA-A--A--AAAAA---A-----A-----  A--AAAAAAA-A--A--AAAAA---AAAAAAA----- | 2.71 |
|  | The length of any line in the MSA | -AAAAAAAAAAAAAAAAA---AAAAAAAAAAA-----  A----AA-AA-AAAA--A---A---A-----AAAAAA  A---AAA-AA-A--A--AAAAA---A-----A-----  A--AAAAAAA-A--A--AAAAA---AAAAAAA----- | 37 |
|  | Length of the largest sequence in the MSA when unaligned | -AAAAAAAAAAAAAAAAA---AAAAAAAAAAA-----  A----AA-AA-AAAA--A---A---A-----AAAAAA  A---AAA-AA-A--A--AAAAA---A-----A-----  A--AAAAAAA-A--A--AAAAA---AAAAAAA----- | 28 |
|  | Length of the shortest sequence in the MSA when unaligned | -AAAAAAAAAAAAAAAAA---AAAAAAAAAAA-----  A----AA-AA-AAAA--A---A---A-----AAAAAA  A---AAA-AA-A--A--AAAAA---A-----A-----  A--AAAAAAA-A--A--AAAAA---AAAAAAA----- | 15 |
|  | The total number of gaps in the MSA | -AAAAAAAAAAAAAAAAA---AAAAAAAAAAA-----  A----AA-AA-AAAA--A---A---A-----AAAAAA  A---AAA-AA-A--A--AAAAA---A-----A-----  A--AAAAAAA-A--A--AAAAA---AAAAAAA----- | 24 |
|  | Number of gaps of length one | -AAAAAAAAAAAAAAAAA---AAAAAAAAAAA-----  A----AA-AA-AAAA--A---A---A-----AAAAAA  A---AAA-AA-A--A--AAAAA---A-----A-----  A--AAAAAAA-A--A--AAAAA---AAAAAAA----- | 6 |
|  | Number of gaps of length two | -AAAAAAAAAAAAAAAAA---AAAAAAAAAAA-----  A----AA-AA-AAAA--A---A---A-----AAAAAA  A---AAA-AA-A--A--AAAAA---A-----A-----  A--AAAAAAA-A--A--AAAAA---AAAAAAA----- | 6 |
|  | Number of gaps of length three | -AAAAAAAAAAAAAAAAA---AAAAAAAAAAA-----  A----AA-AA-AAAA--A---A---A-----AAAAAA  A---AAA-AA-A--A--AAAAA---A-----A-----  A--AAAAAAA-A--A--AAAAA---AAAAAAA----- | 6 |
|  | Number of gaps of length larger than three | -AAAAAAAAAAAAAAAAA---AAAAAAAAAAA-----  A----AA-AA-AAAA--A---A---A-----AAAAAA  A---AAA-AA-A--A--AAAAA---A-----A-----  A--AAAAAAA-A--A--AAAAA---AAAAAAA----- | 6 |
|  | Average size of unique gaps:  $\frac{\sum\text{length of uniquegaps}}{\text{total number of unique gaps}}$ | -AAAAAAAAAAAAAAAAA---AAAAAAAAAAA-----  A----AA-AA-AAAA--A---A---A-----AAAAAA  A---AAA-AA-A--A--AAAAA---A-----A-----  A--AAAAAAA-A--A--AAAAA---AAAAAAA----- | 2.67 |
|  | Total number of unique gaps | -AAAAAAAAAAAAAAAAA---AAAAAAAAAAA-----  A----AA-AA-AAAA--A---A---A-----AAAAAA  A---AAA-AA-A--A--AAAAA---A-----A-----  A--AAAAAAA-A--A--AAAAA---AAAAAAA----- | 12 |
|  | Number of gaps of length one that are in exactly one sequence | -AAAAAAAAAAAAAAAAA---AAAAAAAAAAA-----  A----AA-AA-AAAA--A---A---A-----AAAAAA  A---AAA-AA-A--A--AAAAA---A-----A-----  A--AAAAAAA-A--A--AAAAA---AAAAAAA----- | 1 |
|  | Number of gaps of length one that are in exactly two sequences | -AAAAAAAAAAAAAAAAA---AAAAAAAAAAA-----  A----AA-AA-AAAA--A---A---A-----AAAAAA  A---AAA-AA-A--A--AAAAA---A-----A-----  A--AAAAAAA-A--A--AAAAA---AAAAAAA----- | 1 |
|  | Number of gaps of length one that are in all sequences except one | -AAAAAAAAAAAAAAAAA---AAAAAAAAAAA-----  A----AA-AA-AAAA--A---A---A-----AAAAAA  A---AAA-AA-A--A--AAAAA---A-----A-----  A--AAAAAAA-A--A--AAAAA---AAAAAAA----- | 1 |
|  | Number of gaps of length two that are in exactly one sequence | -AAAAAAAAAAAAAAAAA---AAAAAAAAAAA-----  A----AA-AA-AAAA--A---A---A-----AAAAAA  A---AAA-AA-A--A--AAAAA---A-----A-----  A--AAAAAAA-A--A--AAAAA---AAAAAAA----- | 1 |
|  | Number of gaps of length two that are in exactly two sequences | -AAAAAAAAAAAAAAAAA---AAAAAAAAAAA-----  A----AA-AA-AAAA--A---A---A-----AAAAAA  A---AAA-AA-A--A--AAAAA---A-----A-----  A--AAAAAAA-A--A--AAAAA---AAAAAAA----- | 1 |
|  | Number of gaps of length two that are in all sequences except one | -AAAAAAAAAAAAAAAAA---AAAAAAAAAAA-----  A----AA-AA-AAAA--A---A---A-----AAAAAA  A---AAA-AA-A--A--AAAAA---A-----A-----  A--AAAAAAA-A--A--AAAAA---AAAAAAA----- | 1 |
|  | Number of gaps of length three that are in exactly one sequence | -AAAAAAAAAAAAAAAAA---AAAAAAAAAAA-----  A----AA-AA-AAAA--A---A---A-----AAAAAA  A---AAA-AA-A--A--AAAAA---A-----A-----  A--AAAAAAA-A--A--AAAAA---AAAAAAA----- | 1 |
|  | Number of gaps of length three that are in exactly two sequences | -AAAAAAAAAAAAAAAAA---AAAAAAAAAAA-----  A----AA-AA-AAAA--A---A---A-----AAAAAA  A---AAA-AA-A--A--AAAAA---A-----A-----  A--AAAAAAA-A--A--AAAAA---AAAAAAA----- | 1 |
|  | Number of gaps of length three that are in all sequences except one | -AAAAAAAAAAAAAAAAA---AAAAAAAAAAA-----  A----AA-AA-AAAA--A---A---A-----AAAAAA  A---AAA-AA-A--A--AAAAA---A-----A-----  A--AAAAAAA-A--A--AAAAA---AAAAAAA----- | 1 |
|  | Number of gaps of length larger than three that are in exactly one sequence | -AAAAAAAAAAAAAAAAA---AAAAAAAAAAA-----  A----AA-AA-AAAA--A---A---A-----AAAAAA  A---AAA-AA-A--A--AAAAA---A-----A-----  A--AAAAAAA-A--A--AAAAA---AAAAAAA----- | 1 |
|  | Number of gaps of length three that are in exactly two sequences | -AAAAAAAAAAAAAAAAA---AAAAAAAAAAA-----  A----AA-AA-AAAA--A---A---A-----AAAAAA  A---AAA-AA-A--A--AAAAA---A-----A-----  A--AAAAAAA-A--A--AAAAA---AAAAAAA----- | 1 |
|  | Number of gaps of length three that are in all sequences except one | -AAAAAAAAAAAAAAAAA---AAAAAAAAAAA-----  A----AA-AA-AAAA--A---A---A-----AAAAAA  A---AAA-AA-A--A--AAAAA---A-----A-----  A--AAAAAAA-A--A--AAAAA---AAAAAAA----- | 1 |
|  | Number of positions (**columns**) in the MSA that do not contain any gap. | -AAAAAAAAAAAAAAAAA---AAAAAAAAAAA-----  A----AA-AA-AAAA--A---A---A-----AAAAAA  A---AAA-AA-A--A--AAAAA---A-----A-----  A--AAAAAAA-A--A--AAAAA---AAAAAAA----- | 10 |
|  | Number of positions in the MSA that contain exactly one gap. | -AAAAAAAAAAAAAAAAA---AAAAAAAAAAA-----  A----AA-AA-AAAA--A---A---A-----AAAAAA  A---AAA-AA-A--A--AAAAA---A-----A-----  A--AAAAAAA-A--A--AAAAA---AAAAAAA----- | 2 |
|  | Number of positions in the MSA that contain exactly two gaps. | -AAAAAAAAAAAAAAAAA---AAAAAAAAAAA-----  A----AA-AA-AAAA--A---A---A-----AAAAAA  A---AAA-AA-A--A--AAAAA---A-----A-----  A--AAAAAAA-A--A--AAAAA---AAAAAAA----- | 12 |
|  | Number of positions in the MSA that contain a gap in all sequences except one. | -AAAAAAAAAAAAAAAAA---AAAAAAAAAAA-----  A----AA-AA-AAAA--A---A---A-----AAAAAA  A---AAA-AA-A--A--AAAAA---A-----A-----  A--AAAAAAA-A--A--AAAAA---AAAAAAA----- | 13 |

Table S2: Accuracy results of the classification scheme without each of the summary statistics on the EggNOG dataset ENOG503HQ7D. The overall classification accuracy including all summary statistics was 95%.

| **#** | **Summary statistic excluded from classification** | **Accuracy** |
| --- | --- | --- |
|  | The average length of gaps in the MSA | 95.00% |
|  | The length of any line in the MSA | 93.00% |
|  | Length of the largest sequence in the MSA when unaligned | 94.33% |
|  | Length of the shortest sequence in the MSA when unaligned | 95.00% |
|  | The total number of gaps in the MSA | 91.00% |
|  | Number of gaps of length one | 91.67% |
|  | Number of gaps of length two | 92.33% |
|  | Number of gaps of length three | 91.00% |
|  | Number of gaps of length larger than three | 90.00% |
|  | Average size of unique gaps | 95.67% |
|  | Total number of unique gaps | 94.00% |
|  | Number of gaps of length one that are in exactly one sequence | 94.33% |
|  | Number of gaps of length one that are in exactly two sequences | 94.67% |
|  | Number of gaps of length one that are in all sequences except one | 94.00% |
|  | Number of gaps of length two that are in exactly one sequence | 93.67% |
|  | Number of gaps of length two that are in exactly two sequences | 94.33% |
|  | Number of gaps of length two that are in all sequences except one | 94.33% |
|  | Number of gaps of length three that are in exactly one sequence | 92.67% |
|  | Number of gaps of length three that are in exactly two sequences | 94.67% |
|  | Number of gaps of length three that are in all sequences except one | 94.33% |
|  | Number of gaps of length larger than three that are in exactly one sequence | 95.00% |
|  | Number of gaps of length three that are in exactly two sequences | 94.33% |
|  | Number of gaps of length three that are in all sequences except one | 94.33% |
|  | Number of positions (**columns**) in the MSA that do not contain any gap. | 94.33% |
|  | Number of positions in the MSA that contain exactly one gap. | 95.00% |
|  | Number of positions in the MSA that contain exactly two gaps. | 95.33% |
|  | Number of positions in the MSA that contain a gap in all sequences except one. | 94.33% |

| (a) | (b) |
| --- | --- |
| 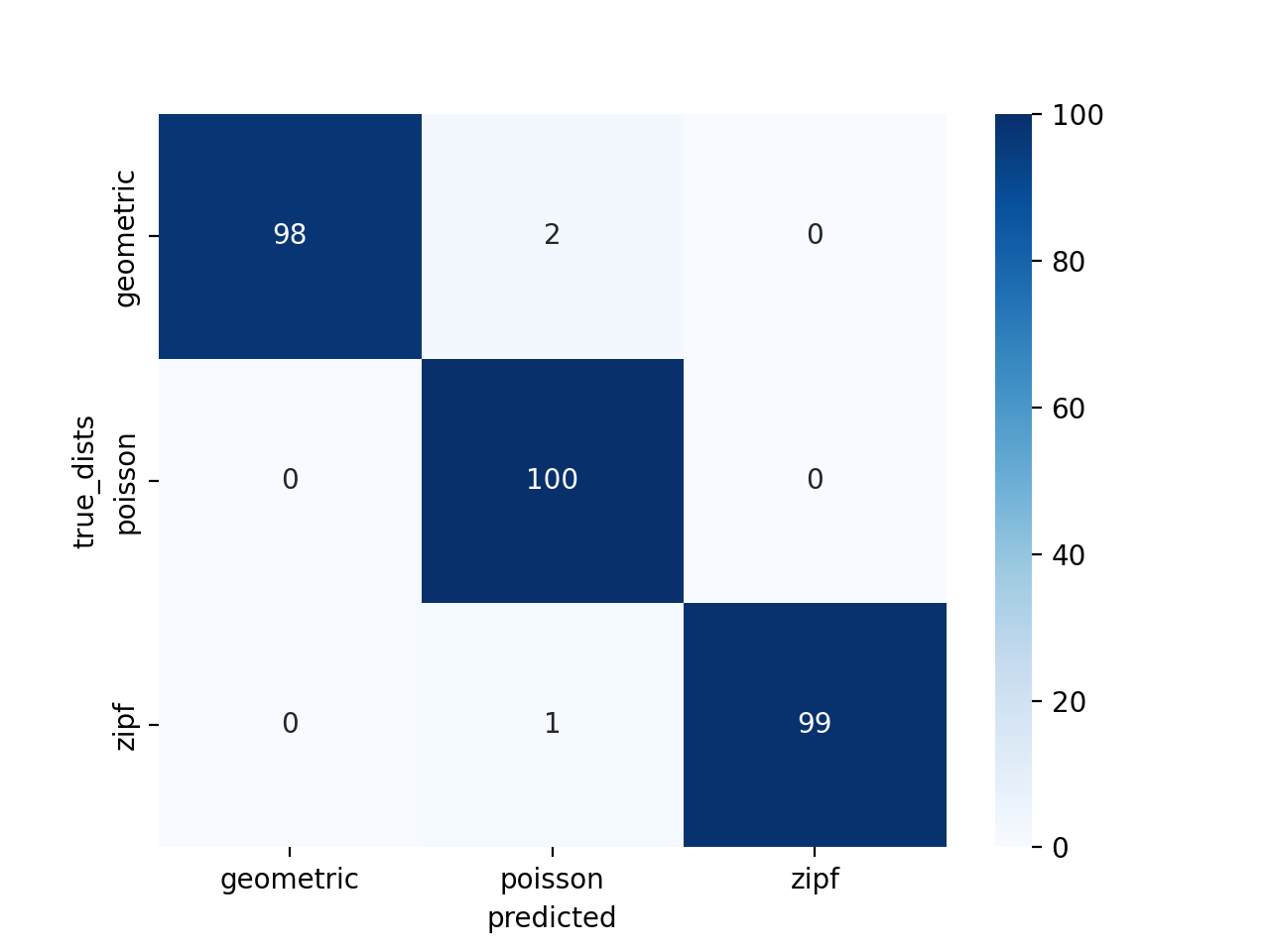 | 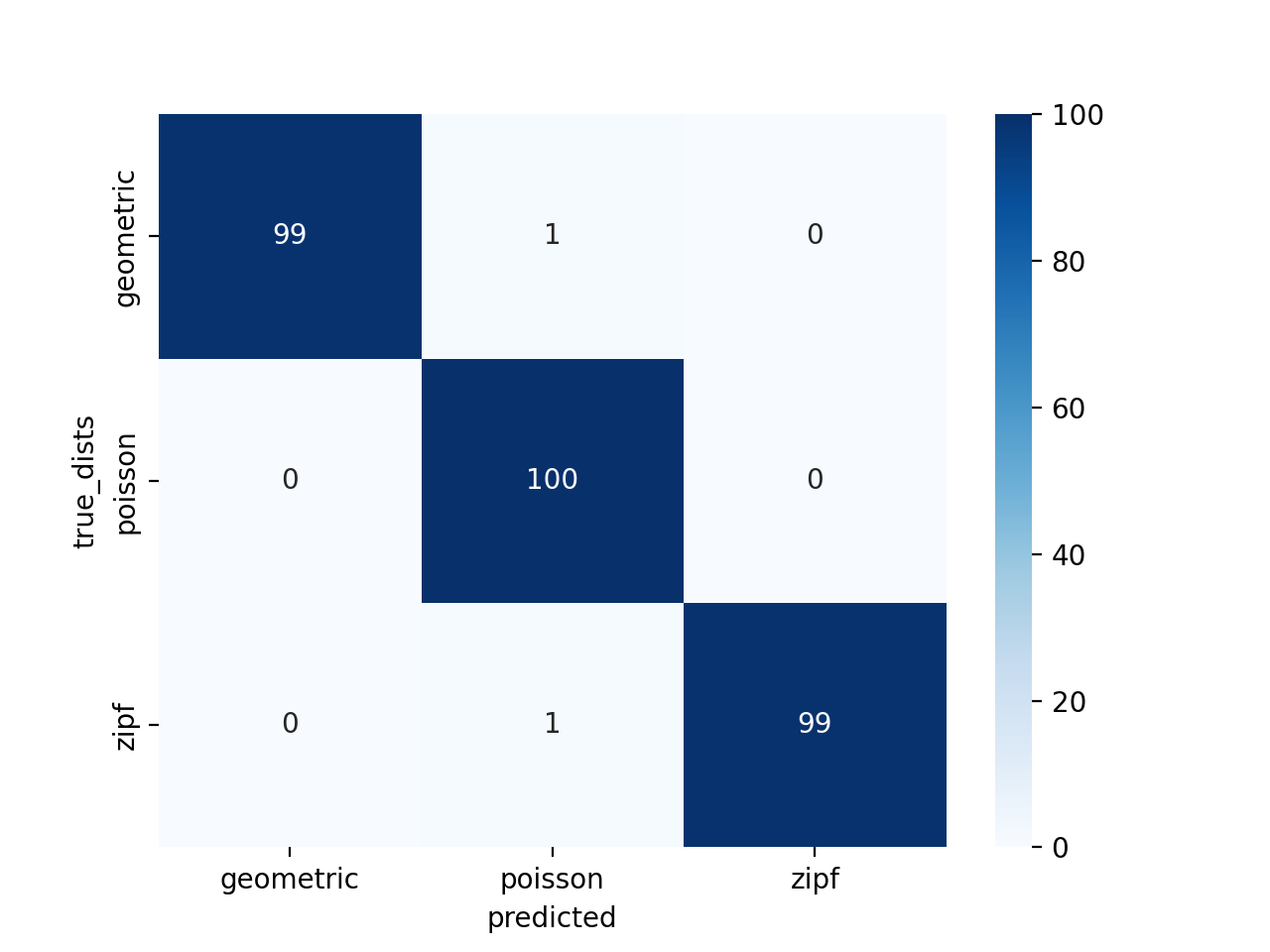 |
| (c) | (d) |
| 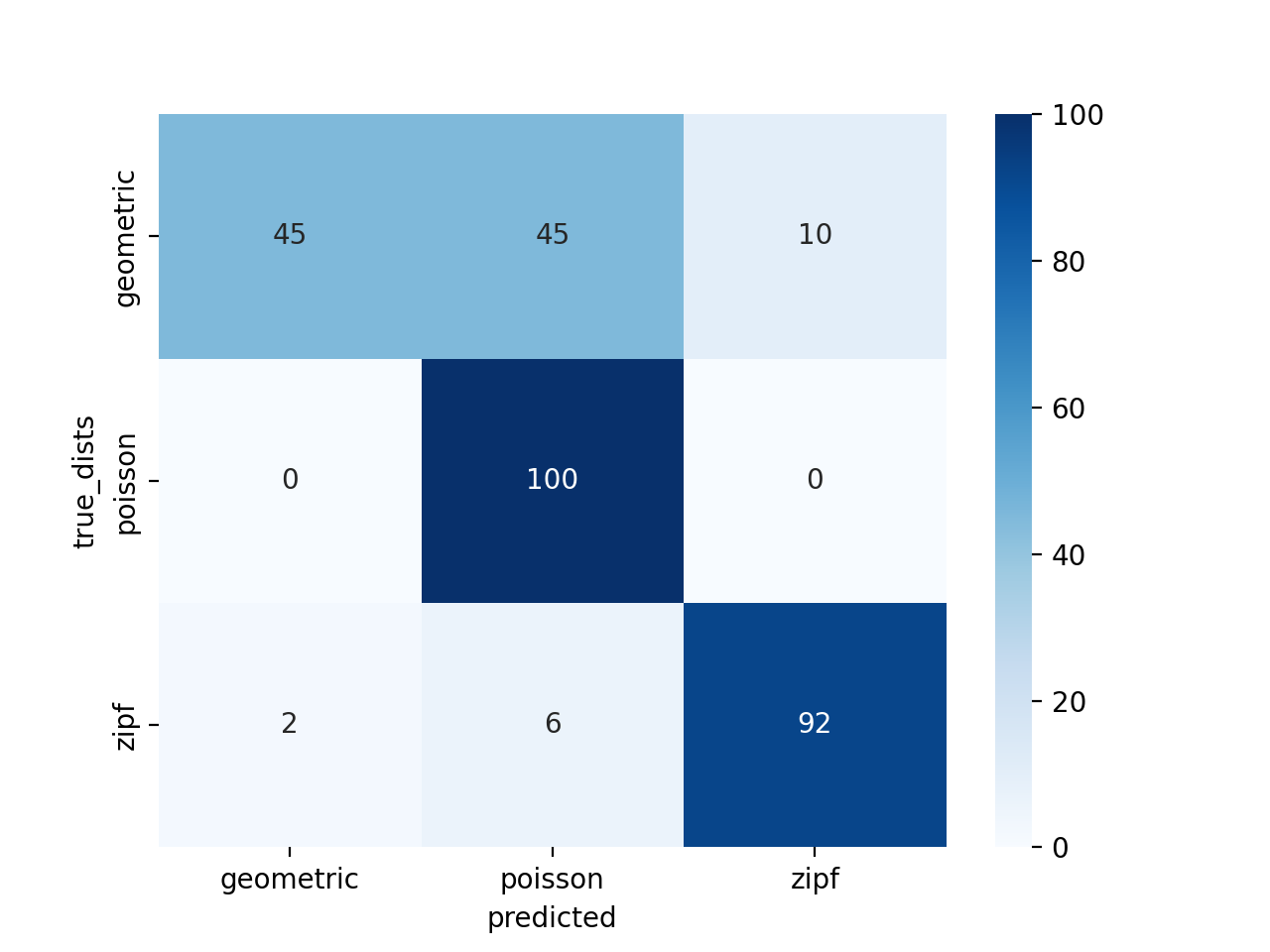 | 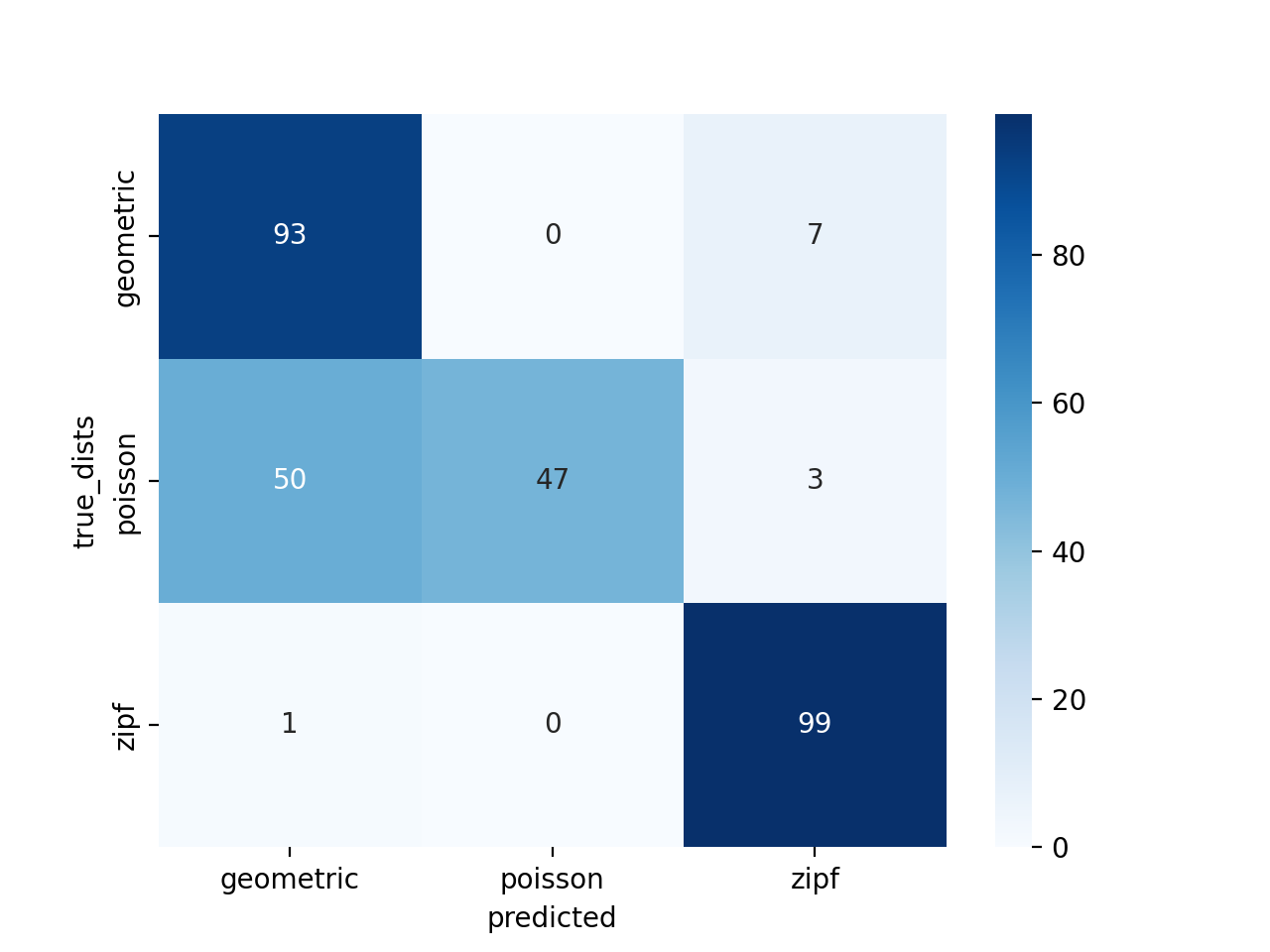 |

Figure S1: Confusion matrices of the classification results on simulation with extreme indel parameters on the EggNOG dataset ENOG503HQ7D: (a) very high insertion and deletion rates; (b) very low insertion and deletion rates; (c) very high length parameters of insertion and deletion; (d) very low length parameters of insertion and deletion.


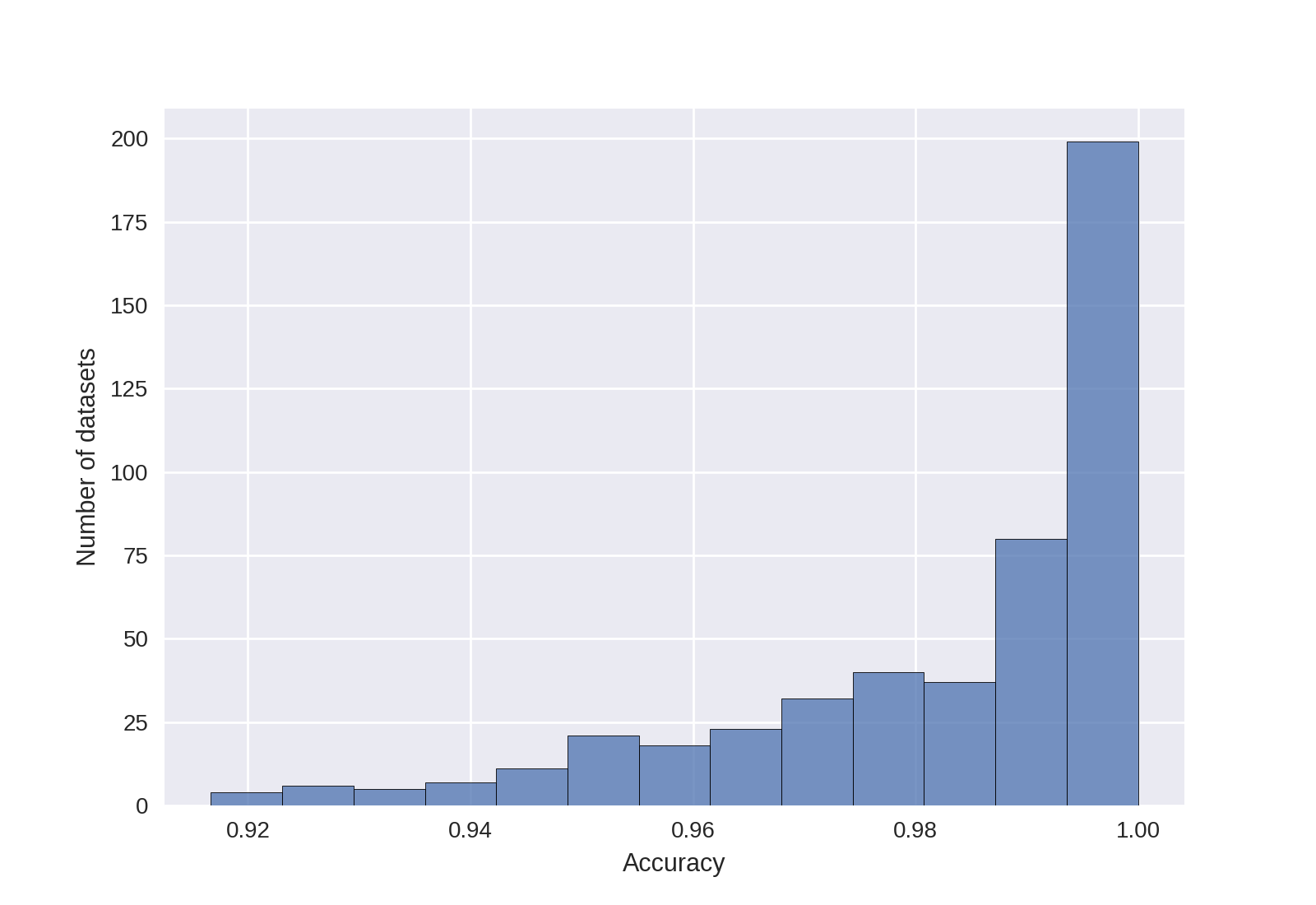
Figure S2: Histogram of the accuracies for simulations derived using phylogenetic trees taken from the EggNOG database and the YIDB database, with $n_{i}=100$ and $n_{s}=3,000,000$.

| (a) | (b) |
| --- | --- |
| 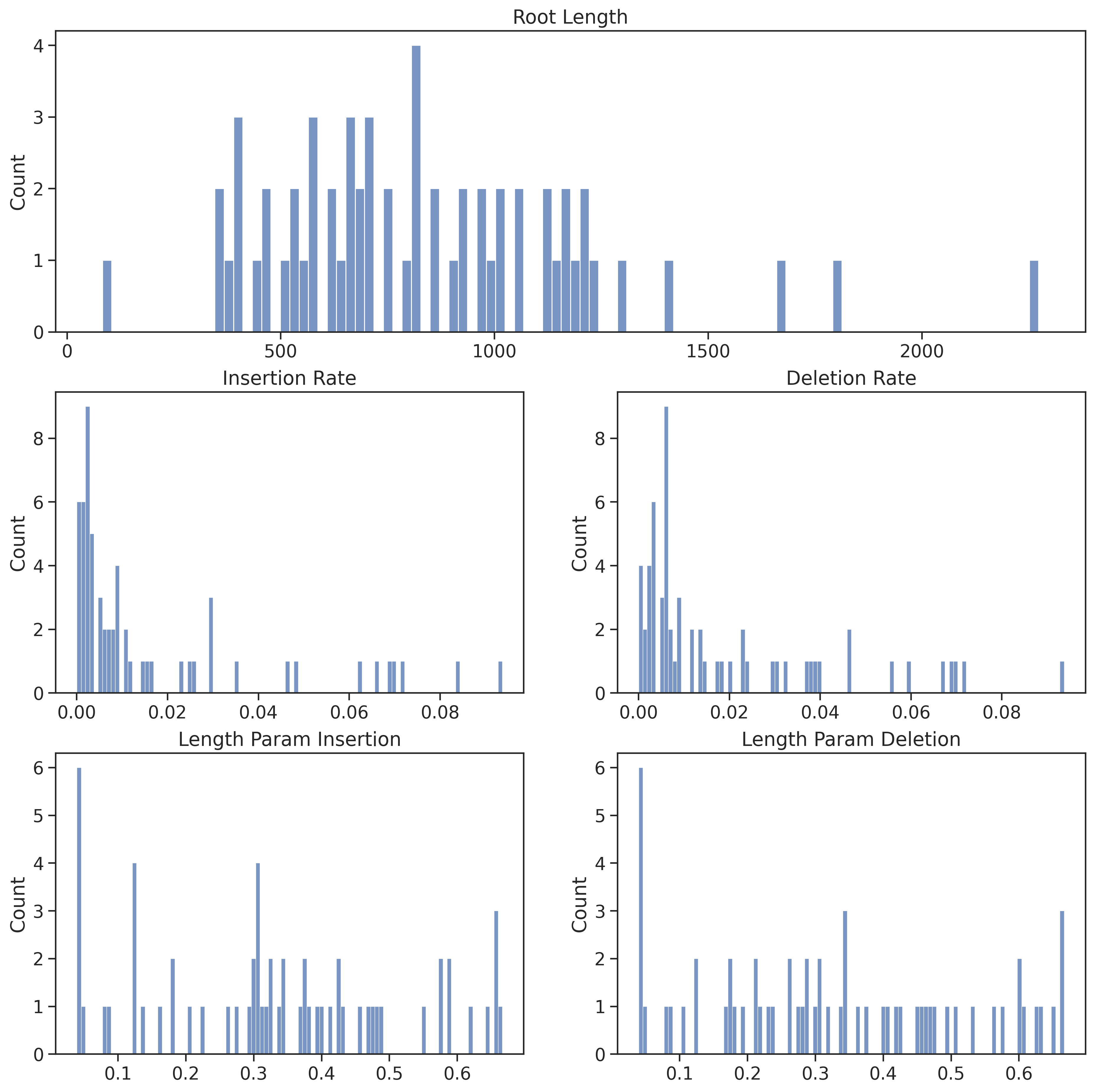 | 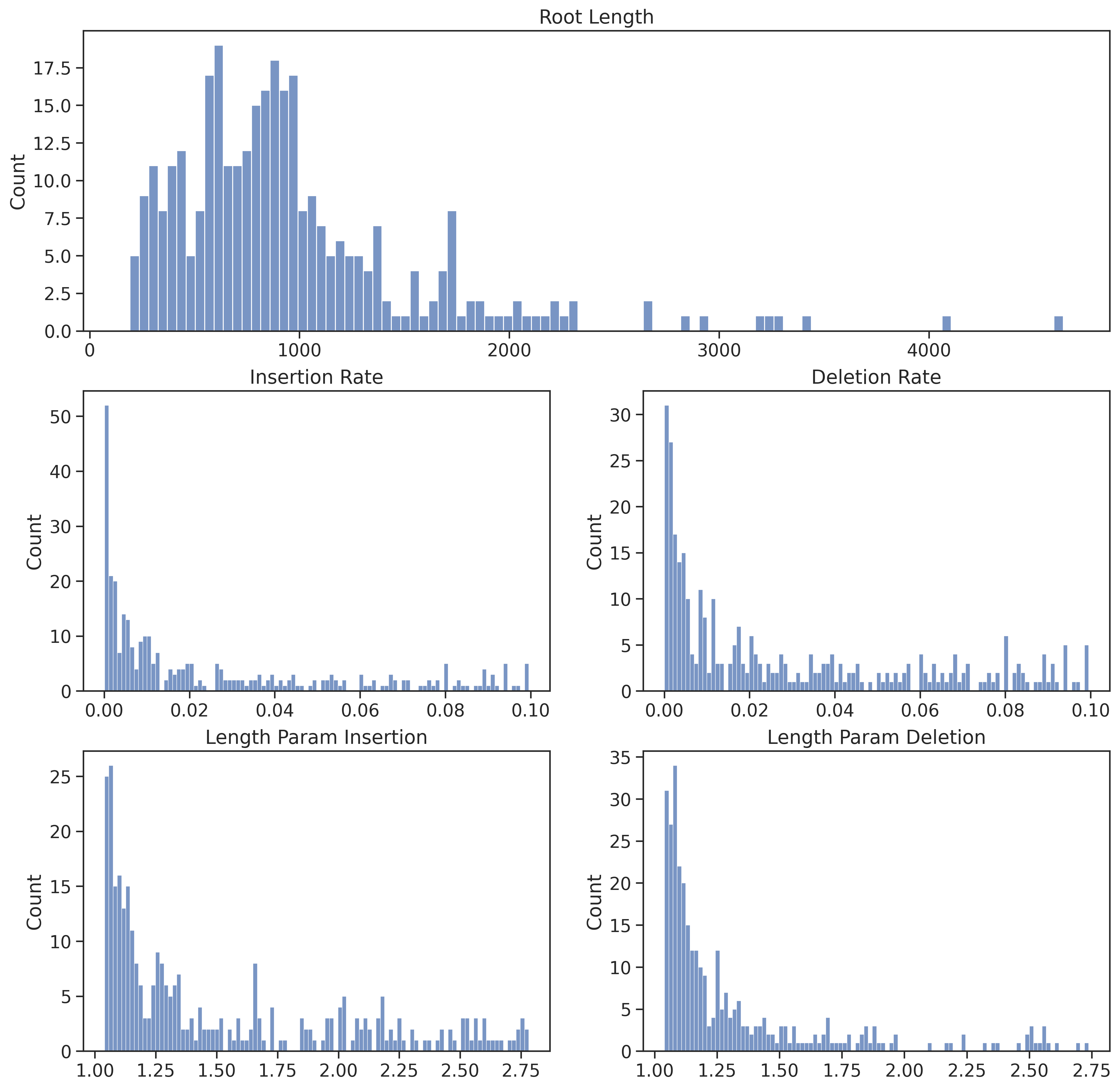 |
| (c) | (d) |
| 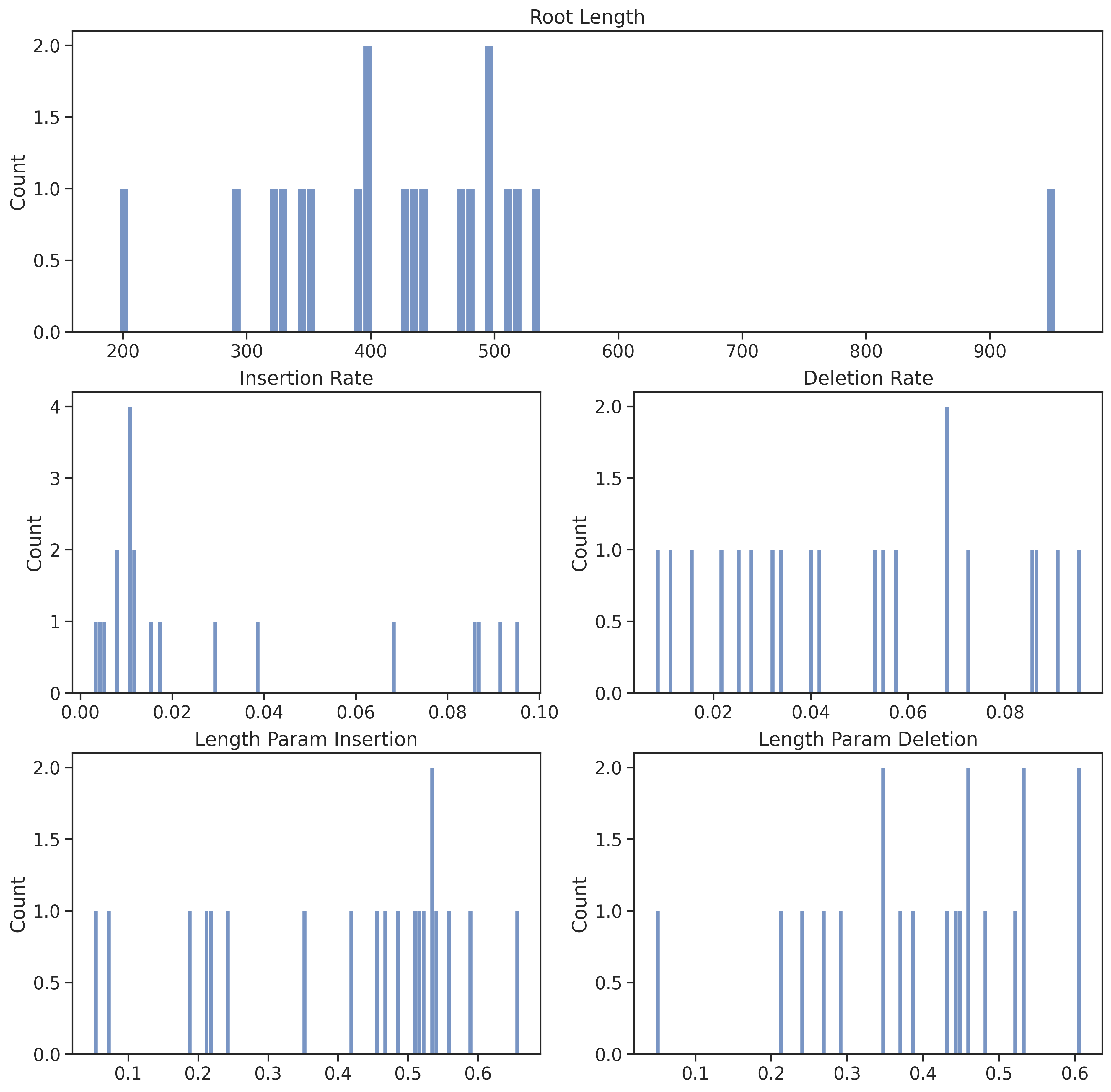 | 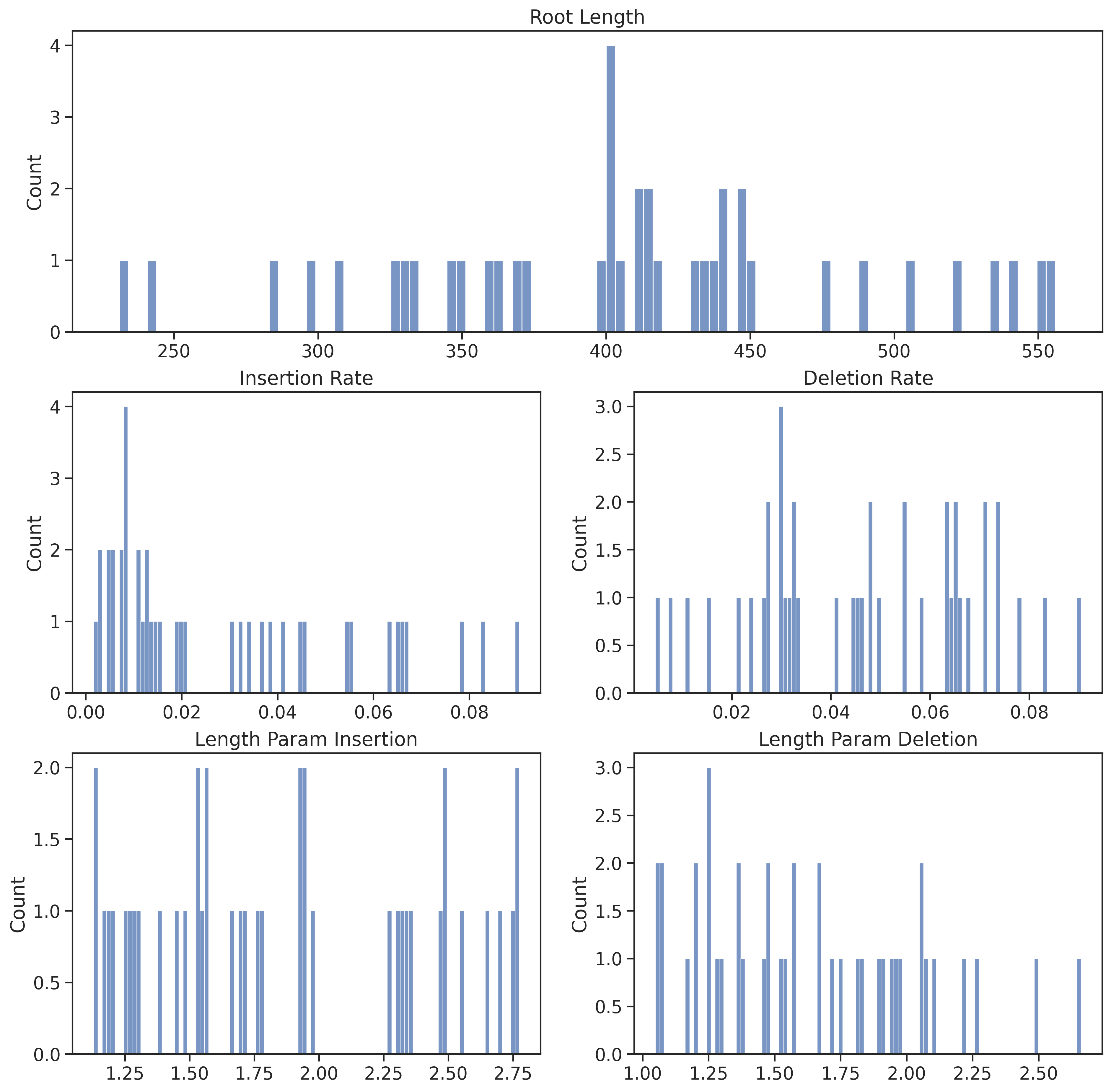 |

Figure S3: Posterior parameter distributions for Zipf and geometric model parameters across datasets: (a) Posterior distributions for EggNOG datasets that were classified as geometric; (b) Posterior distributions for EggNOG datasets that were classified as Zipf; (c) Posterior distributions for YIDB datasets that were classified as geometric; (d) Posterior distributions for YIDB datasets that were classified as Zipf.


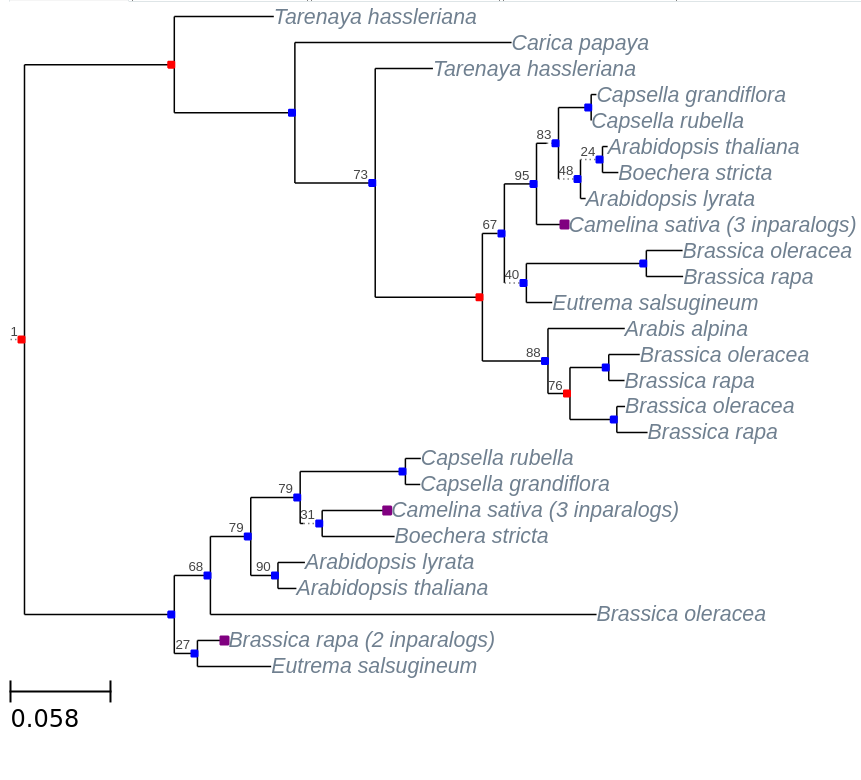
Figure S4: EggNOG dataset ENOG503HQ7D phylogenetic tree visualization.

| (a) | (b) |
| --- | --- |
| 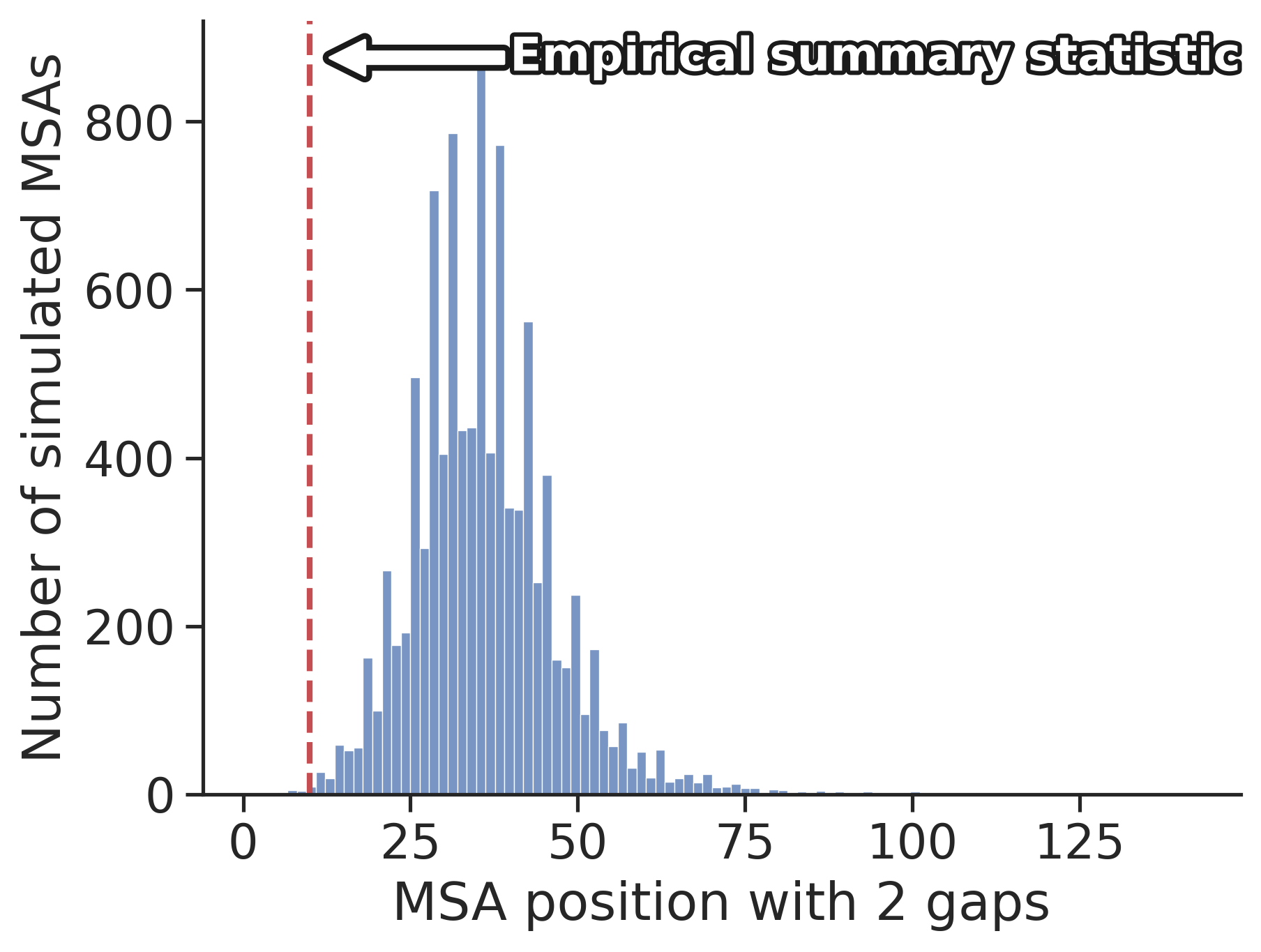 | 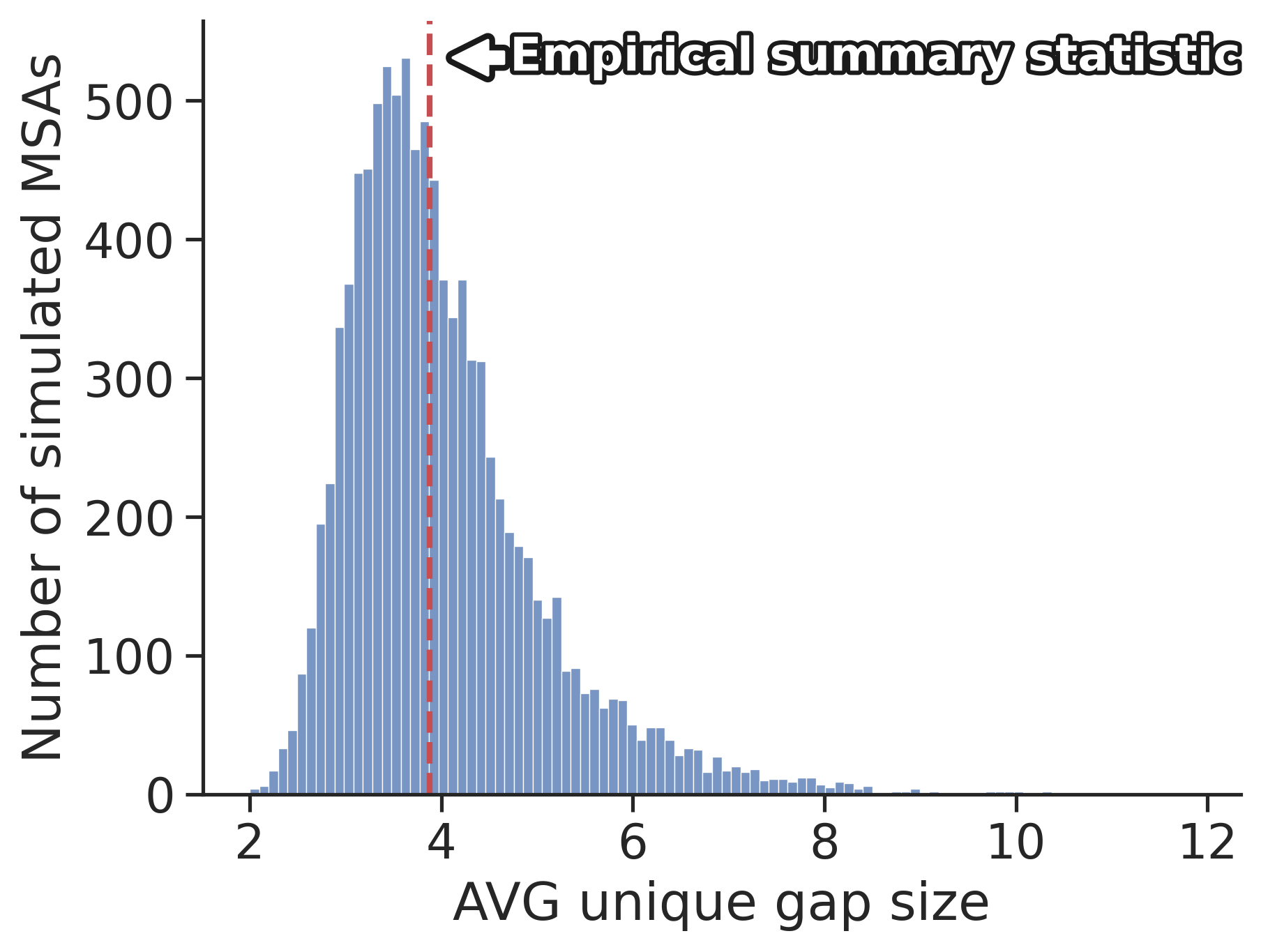 |

Figure S5: The distribution of two specific summary statistics in MSAs simulated using the top 50 priors parameters of the geometric distribution model for the EggNOG dataset 3WUBT. (a) The empirical summary statistics lies outside the 95^th^ percentile when considering the number of positions within the MSA that contain only two gaps (summary statistic 26). (b) The empirical summary statistics lies inside the 95^th^ percentile when considering the average length of unique gaps within the MSA (summary statistic 10).

| 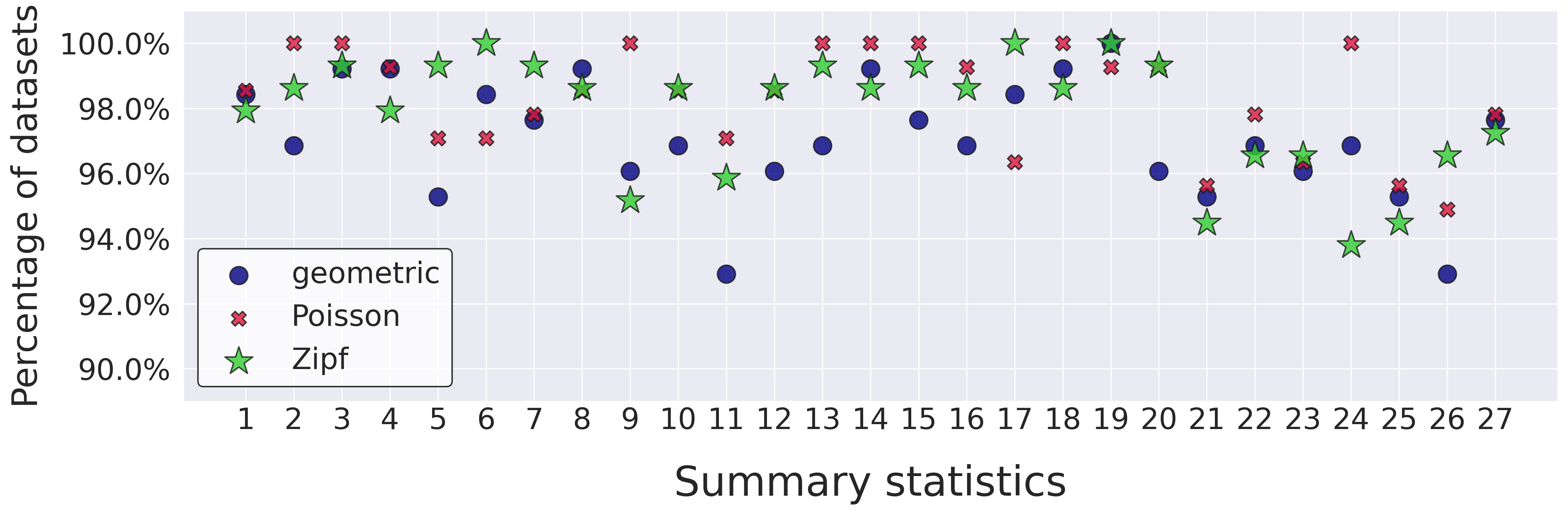 |
| --- |

Figure S6: Summary of the posterior predictive analysis for each of the summary statistics, on simulated data based on 416 trees from the EggNOG database.

The blue dots represent datasets which were classified as geometric, the green stars datasets which were classified as Zipf, and the red crosses which were classified as Poisson.

| 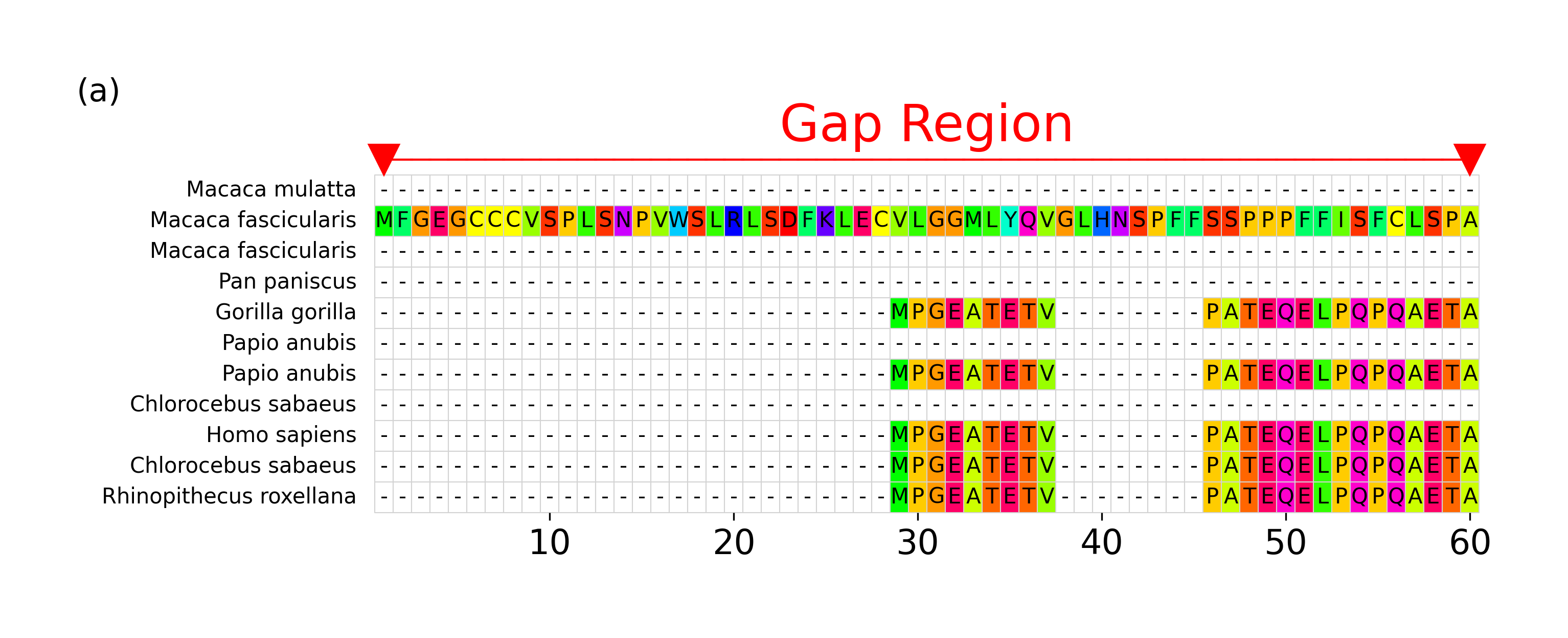 |
| --- |
| 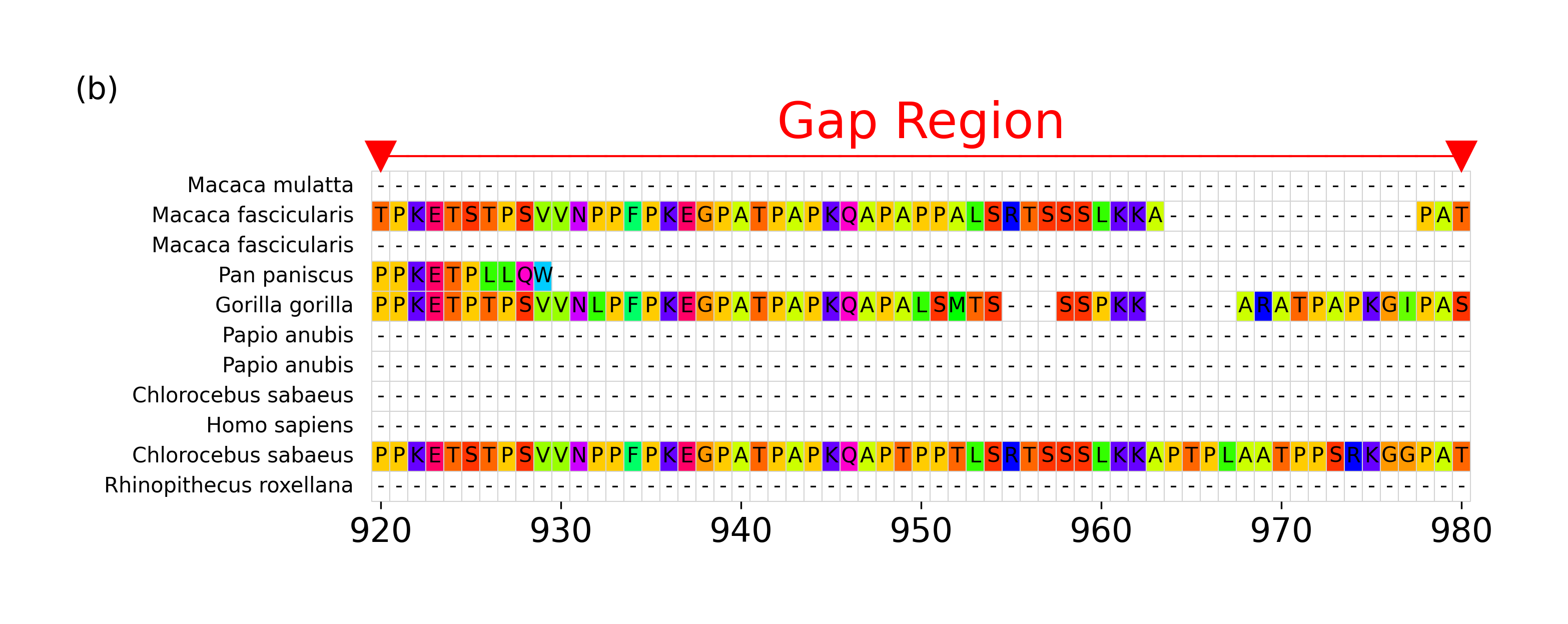 |
| 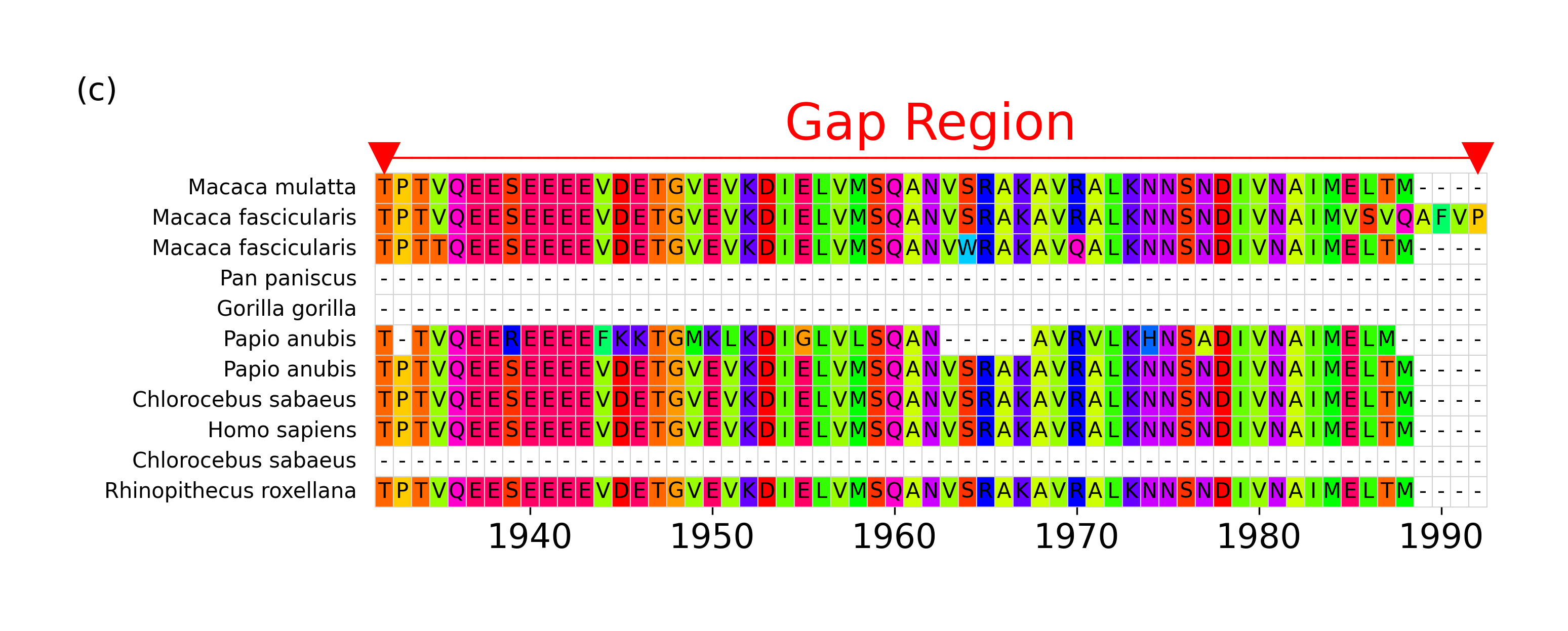 |

Figure S7 An example of an empirical alignment that yielded a low AM score probably due to poor annotation. Visualization of the ENOG504MRKW dataset at three different intervals within the MSA, the MSA has 1992 positions: (a) Positions 1-60; (b) Positions 920-980; (c) Positions: 1932-1992. In addition to these positions, very large gap blocks are present along the entire alignment, causing many summary statistics to be skewed, i.e. the shortest sequence within the MSA is of length 111, while the longest is of length 1,958. The average gap size is 169.5 with 75 total gaps.


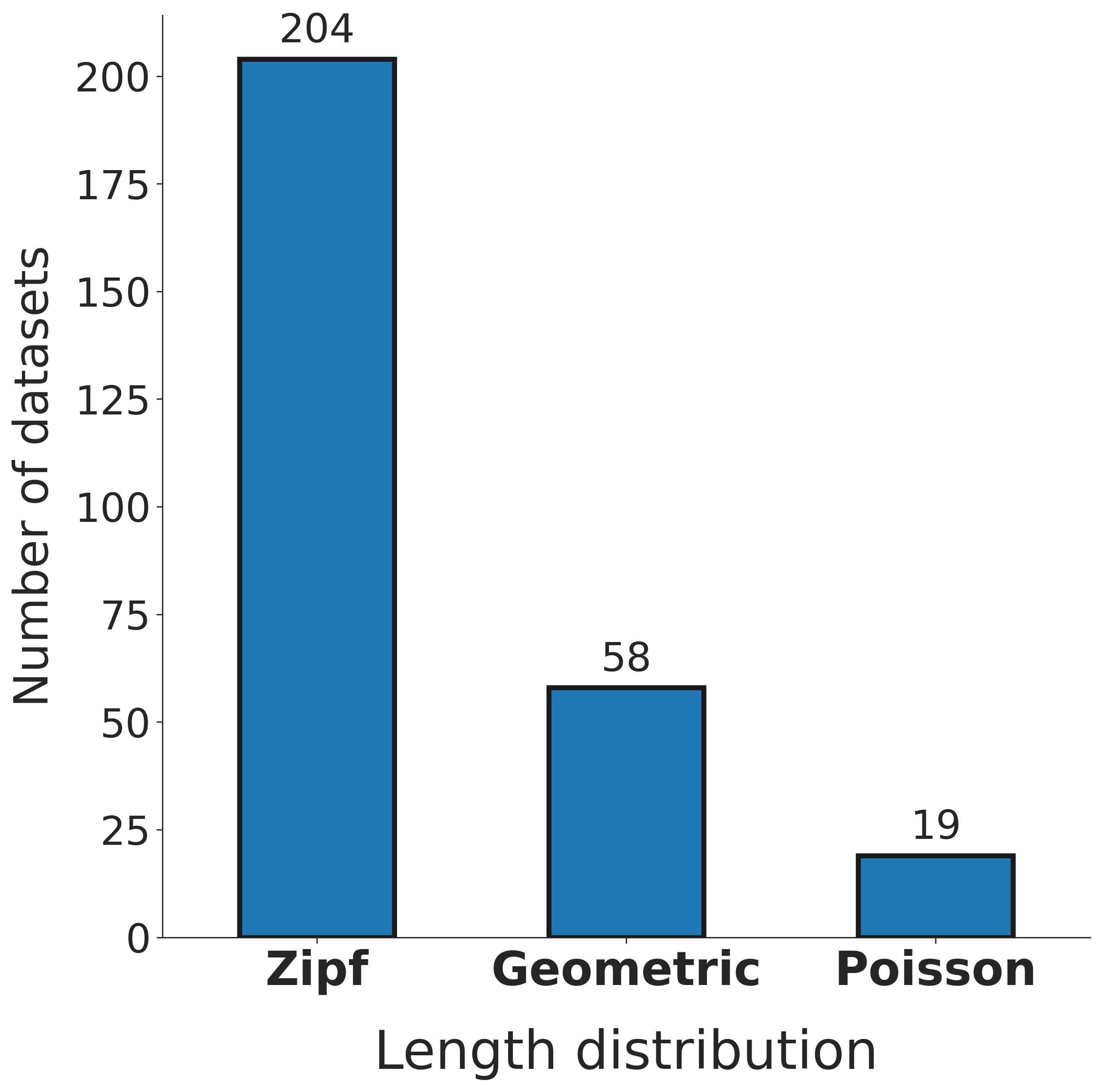
Figure S8: Histogram of classified length distributions: EggNOG datasets with an AM score higher than 0.8.

# Methods:

## ABC threshold selection

In the classic ABC rejection scheme, the subset used for inferring the posterior distribution of the model parameters is chosen by taking those simulations for which the distance between their vectors of summary statistics and the vector of summary statistics derived from the empirical dataset is below a given threshold 𝜀. Since this method has an unbounded running time, we predetermined the number of simulations, *n_i_*, in the inference subset as was done by Beaumont et al. (2002). The inference accuracy depends on *n_i_*, and the total number of simulations performed, *n_s_*. We used simulation-based tests to find the *n_s_* and *n_i_* values that best balance accuracy and computation time. We ran our classification scheme with different values of *n_s_*, and for each such value of *n_s_*, we tested different values of *n_i_*. Our results indicated that using *n_i_*=100 with *n_s_*=3,000,000 (note that this *n_s_* value reflects running 500,000 simulations for each model) balances well accuracy with running time, and we thus used these values for all subsequent analyses. Indeed, on simulated dataset, the average accuracy of our inference scheme was higher than 98% (see Results).

**MSA bias correction**

To correct the bias that may stem from alignments errors, we simulated, for a given dataset, 500 MSAs using the same priors and settings as we simulate the simulations. The simulated MSAs are exact, and we would like to learn the transformation between the vectors of summary statistics computed based on the exact MSA and those computed following re-alignment of the simulated sequences with MAFFT (Katoh and Standley, 2013). To reduce the running time, for most of the ABC analyses we simulated only indel events and ignored substitutions, since the character identities (nucleotides or amino-acids) are irrelevant for computing the summary statistics. However, for the bias correction we need character assignments because we unalign the exact MSAs and then realign the sequences using MAFFT. To this end, we used INDELIBLE (Fletcher and Yang, 2009) to generate only substitution events (setting the indel rate to be zero). Next, we combined the indel events from our simulator and the substitution events from INDELIBLE, thus obtaining an exact MSA that has both characters and indels. Afterward, we unaligned these exact MSAs and realigned them using MAFFT. We extracted the summary statistics vectors from the exact and the realigned MSAs and used Lasso regression (Tibshirani, 1996) to learn the transformation between the corresponding vectors. Lastly, we applied the transformation on the summary statistics vectors to compensate for the bias of the empirical MSAs.
